# A systematic review finds Core Outcome Set uptake varies widely across different areas of health

**DOI:** 10.1016/j.jclinepi.2020.09.029

**Published:** 2021-01

**Authors:** Karen L. Hughes, Mike Clarke, Paula R. Williamson

**Affiliations:** a MRC North West Hub for Trials Methodology Research, Department of Health Data Science, University of Liverpool, Block F Waterhouse Building, 1-5 Brownlow Street, Liverpool L69 3GL, United Kingdom; b Centre for Public Health, Institute of Clinical Sciences, Block B, Queen's University Belfast, Royal Victoria Hospital, Grosvenor Road, Belfast BT12 6BA, United Kingdom

**Keywords:** Core outcome set, COS, Uptake, Clinical trials, Systematic reviews, Research waste

## Abstract

**Objective:**

The aim of our review was to bring together studies that had assessed the uptake of core outcome sets (COS) to explore the level of uptake across different COS and areas of health.

**Study Design and Setting:**

We examined the citations of 337 COS reports to identify studies that had assessed the uptake of a particular COS in randomized controlled trials (RCTs) or systematic reviews (SRs).

**Results:**

We identified 24 studies that had assessed uptake in RCTs and two studies that had assessed uptake in SRs. The studies covered a total of 17/337 (5%) COS. Uptake rates reported for RCTs varied from 0% of RCTs (gout) to 82% RCTs (rheumatoid arthritis) measuring the full COS. Studies that assessed uptake of individual core outcomes showed a wide variation in uptake between the outcomes. Suggested barriers to uptake included lack of validated measures, lack of patient and other key stakeholder involvement in COS development, and lack of awareness of the COS.

**Conclusions:**

Few studies have been undertaken to assess the uptake of COS in RCTs and SRs. Further studies are needed to assess whether COS have been implemented across a wider range of disease categories and to explore the barriers and facilitators to COS uptake.

**What is new?**

**Key findings**
• Few studies have assessed core outcome set (COS) uptake (17/337 (5%) COS assessed).
• There is wide variation in COS uptake across different health areas.

**What this adds to what is known?**
• This review will serve as a benchmark for comparing uptake going forward.

**What is the implication, what should change now?**
• Barriers and facilitators to COS uptake should be explored.

## 1 Introduction

In 2014 Gargon et al., under the auspices of the COMET (Core Outcome Measures for Effectiveness Trials) Initiative, published a systematic review bringing together studies that had made recommendations about which outcomes to measure in clinical trials of specific health conditions [1]. Such recommendations, known as Core Outcome Sets (COS), are defined by the COMET Initiative as “an agreed standardized set of outcomes that should be measured and reported, as a minimum, in all clinical trials in specific areas of health or health care.” Gargon’s review identified 250 publications relating to 198 COS that had been developed up to August 2013. Following the initial review, updates have been published annually [[2], [3], [4], [5], 6]] with the latest bringing the number of COS to 364, described in 403 publications, up to the end of 2018. As of March 2020, a further 267 COS in development were registered in the database maintained by the COMET Initiative (www.comet-initiative.org/Studies).

The development of COS tackles problems with outcomes in trials, including lack of standardization, which hampers evidence synthesis [7], outcome reporting bias [8], and relevance of the outcomes [9]. Through the involvement of key stakeholders and the use of consensus methods to agree on the set of core outcomes, COS can provide the consistency and relevance needed to address the problems with outcomes in trials and other research. However, patients, healthcare professionals, and all other end users of trial results will only benefit from COS if researchers choosing outcomes for trials include them in their studies. In addition, there is a danger that the continuous development of COS, without uptake, will itself result in research waste, contrary to the rationale for COS.

It is, therefore, important that COS developers consider what steps they can take to increase uptake of their COS and monitor its use to establish whether uptake is being achieved. Assessing the uptake of COS in clinical trials or systematic reviews of trials offers COS developers the opportunity to revisit their strategies for promoting uptake where this is found to be low. An assessment of uptake can also allow developers to review the relevance of their COS. For example, if outcomes in the COS are not being used, or trials are consistently measuring an outcome that does not appear in the COS, an update may be suggested.

As the number of COS continues to grow, we did this review to identify studies that have evaluated the uptake of a COS, explore the level of uptake across different areas of health, and review the methods used to assess uptake.

## 2 Methods

The protocol is available at http://www.comet-initiative.org/Studies/Details/1575.

### 2.1 Identification of relevant studies

#### 2.1.1 Citation analysis

Studies were identified by reviewing the citations received by articles reporting a COS published between 1981 and July 2016. The rationale for this method was that a study assessing uptake of a COS should cite the publication reporting that COS. We set this timeframe because the first COS article that we are aware of was published in 1981 and we started accessing citation reports in July 2018. A cut-off date of July 2016 for the publication of the COS was likely to allow sufficient time for the COS to be cited in an uptake study. We included 337 COS publications identified from the COMET Initiative’s systematic reviews that had been published at the time of data collection [[1], [2], [3], [4], [5]] (Supplementary File A for included COS publications). We accessed the citation reports for each COS publication using Scopus, which has been found to include more articles for citation analysis than Web of Science and is more up to date than Google Scholar [10].

#### 2.1.2 Scopus alerts

For ensuring that this review remained current, an alert was set in Scopus to capture studies of COS uptake published after July 2018 that would not appear in our citation search.

### 2.2 Inclusion and exclusion criteria

Studies were included if they had assessed the uptake of the outcomes recommended by the COS, either individually or as a full set, by randomized controlled trials (RCTs) or systematic reviews (SRs). If studies had assessed uptake in additional types of study, e.g., observational studies, we only included data for the RCTs and SRs in our results. We included studies that had reported data that allowed the COS uptake rate to be calculated, even if COS uptake was not the main purpose of the study. Studies were ineligible if they had assessed uptake of outcome measures without an assessment of the recommended outcome domains. Studies were excluded if they had not assessed uptake of all of the outcomes in the COS, e.g., if they had only assessed uptake of the patient-reported outcomes recommended by the COS, to ascertain the level of compliance with the full recommendations of the COS and make comparisons across health areas.

### 2.3 Selecting studies for inclusion

The references and abstracts of all publications that had cited the 337 COS articles were identified using Scopus and exported into Microsoft Excel. If a reference appeared more than once in the Excel file, because the publication had cited more than one COS article, and therefore, appeared in more than one COS article’s citation report, we removed the duplicate references. We searched the titles of each citing publication using keywords relating to COS and uptake to identify possible studies of COS uptake (Supplementary File B for keywords). The resulting titles were assessed, followed by a review of the abstract for those judged to be possible studies of COS uptake. Full texts were examined for those where it was judged from the abstract that the publication might be reporting an assessment of the uptake of a COS or where an abstract was not available. The references in each of the eligible studies were checked for further studies of COS uptake.

### 2.4 Checking for correct exclusion

To confirm the assessment of titles by the first reviewer (KH), a second reviewer (PW) independently assessed 50 titles. As a complete agreement was reached on inclusion and exclusion of articles at this stage, KH completed the rest of the title assessments. PW reviewed 20 abstracts and agreed with KH’s assessment, who then completed this stage. The full texts of 10 articles excluded at the title stage and 20 articles excluded at the abstract stage were checked by KH for correct exclusion.

### 2.5 Data extraction

For each eligible study, the following data were extracted and recorded in a data collection form: disease category, disease name, the scope of the uptake study, the period covered by the assessment, number of RCTs/SRs assessed, % RCTs/SRs that measured the full COS and/or % RCTs/SRs that measured each individual outcome in the COS, the method used to assess uptake and suggested barriers and facilitators for uptake. The scope was defined in terms of the population with the health condition and/or intervention type for which RCTs/SRs were identified and assessed for COS uptake.

### 2.6 Data analysis

The results of the review are presented descriptively. We did not carry out any statistical analyses to synthesize the data.

## 3 Results

### 3.1 Studies identified

**Fig. 1:**
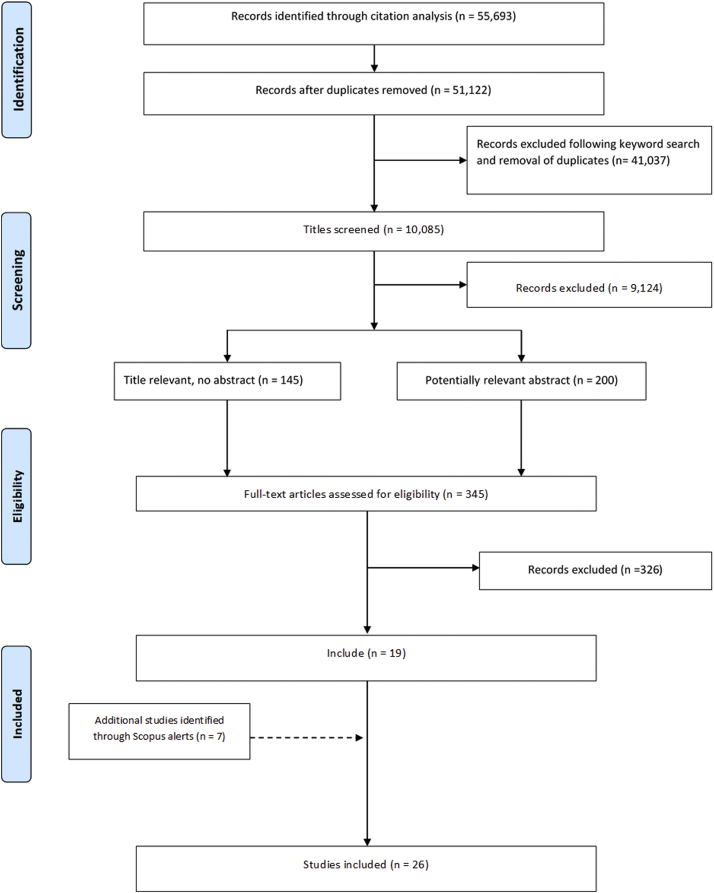
Identification of studies.

The 337 COS publications received a combined total of 55,693 citations with 51,122 remaining after the duplicates had been removed. The titles of 10,085 of the citing articles contained at least one of the keywords. Following the screening of titles and abstracts, 345 full texts were examined, including articles that had no abstract, leading to the identification of 19 studies of COS uptake (Figure 1). A further seven studies were identified via Scopus alerts. We did not identify any additional studies after checking the references of the included studies. Four studies were excluded because they did not assess the uptake of all outcomes that were recommended by the COS. One of these studies assessed uptake of a resource use domain only, while another assessed only uptake of the patient-reported outcomes recommended by the COS. A third study focused on measurement instruments and included an assessment of uptake of some, but not all, COS outcomes and the final study assessed the uptake of a selection of outcomes from a COS that is made up of 48 recommendations. Supplementary File C shows the references of all included studies and the COS they assessed. The 26 studies assessed uptake for a total of 17 COS, with five COS being assessed by more than one study. Thus, we found that 17/337 (5%) COS had been assessed for uptake.

### 3.2 Description of studies

**Fig. 2:**
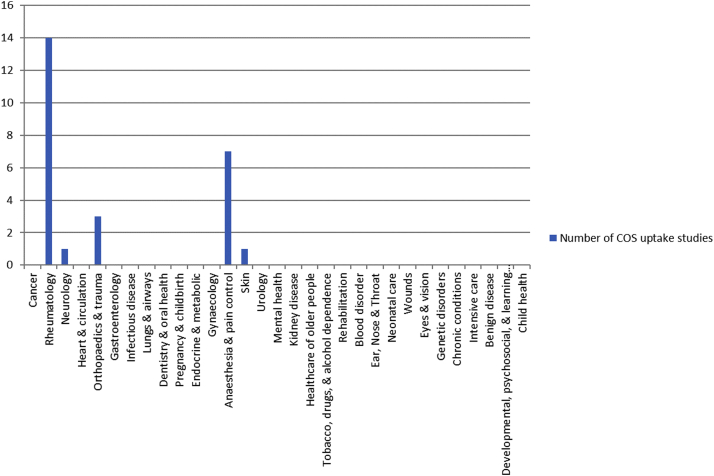
COS uptake studies by disease category.

Twenty-four studies assessed uptake in RCTs, and two studies assessed uptake in SRs (Table 1). The COS assessed were published between 1982 and 2014 and recommended between one and 19 outcomes, with most of them (*n* = 12; 71%) comprising of seven outcomes or fewer (Table 2). The studies assessed between eight and 382 RCTs, and the two assessing SRs included 48 and 90. The 26 studies covered five of 31 disease categories where COS have been developed (Figure 2). Just over half of the studies (*n* = 14) assessed uptake of a rheumatology COS. The other studies assessed uptake of COS developed in the categories of anesthesia and pain control (*n* = 7), orthopedics and trauma (*n* = 3), neurology (*n* = 1), and skin (*n* = 1).

**Table 1:** Studies assessing uptake of COS in RCTs and SRs

COS disease name	Scope of uptake study	Period assessed for uptake of COS	No. RCTs assessed	% RCTs measuring each COS outcome	% RCTs measuring full **COS**
Psoriatic arthritis	Psoriatic arthritis^A4^	2006 – 2010	17	77, 71, 59, 53, 47, 47	24
	Psoriatic arthritis^A13^	2010 – 2015	22	100, 95, 91, 86, 82, 77	59
Knee, hip, and hand	Trapeziometacarpal osteoarthritis^A5^	- 2010	316^a^	96, 94, 67, 59, 4^b^	-
osteoarthritis	Total knee anthroplasty^A16^	- 2014	30	93, 27, 10^c^	7
	Hip or knee osteoarthritis^A23^	1997 – 2017	382	95, 86, 75, 48	45
	Osteoarthritis^A25^	2012 – 2017	334	97, 84, 17, 30	14
Rheumatoid arthritis	DMARD therapy for rheumatoid arthritis^A1^^d^	1986 – 1990	32	100, 91, 91, 91, 91, 73, 73, 64, 55, 55 100, 91, 91, 91, 73, 55, 27	- -
	Rheumatoid arthritis^A2^	2005 – 2007	50^e^	-	82
	Rheumatoid arthritis^A6^	- 2009	350		60-70^f^
	Rheumatoid arthritis^A17^	2002 – 2016	143	-	81
	Rheumatoid arthritis^A22^	2009 - 2019	197	-	Just over 80
Ankylosing spondylitis	Ankylosing spondylitis/axial spondyloarthritis^A7^	- 2013	99	92, 84, 77, 51, 46, 44^g^ 97, 97, 92, 84, 82, 79, 68, 63, 16^h^	20
Acute and chronic	Acute gout^A8^	- 2011	77^i^	99, 57, 51, 32, 5	-
gout	Acute and chronic gout^A11^	- 2013	38^j^ 30^j^	87, 79, 71, 29, 8, 80, 73, 70, 10, 7, 3, 0, 0, 0	5 0
Chronic pain	Cognitive and/or behavioral treatment^A3^	- 2010	60	94, 83, 12 domains >40, 5 domains 0	-
	Acceptance and Commitment Therapy^A9^	1999 - 2014	10	90, 90, 80, 70, 10, 10	-
	Burning mouth syndrome^A21^	1994 – 2017	36	100, 97, 78, 33, 28, 22	11
	Opioids for chronic non-cancer pain^A10^	- 2012	156	99, 94, 76, 46, 43, 31, 28, 19, 7	-
Pediatric acute and chronic pain	Postoperative pain management^A18^	- 2017	337	93, 83, 21, 16, 15, 15	-
Fall injury	Fall prevention in older people^A14^	2005 – 2015	34	94, 47, 24, 24, 21	3
Spinal cord injury	Anticholinergic therapy for neurogenic bladder in SCI^A15^	1946 – 2015	14	3	3
Hip fracture	Hip fracture^A24^	1997 – 2018	311	47, 46, 41, 37, 29	12
Peripheral neuropathy	Multifocal motor neuropathy^A12^	1995 – 2014	8	100, 100, 13	13
Eczema	Atopic eczema treatments^A26^^k^	2005 – 2018	177	-	25%^l^ 33%^m^
			No. SRs assessed	% SRs measuring each COS outcome	% SRs measuring full COS
Chronic pain	Neuropathic pain conditions^A19^	- 2015	90	94, 84, 53, 50, 49, 29	10
Pediatric acute and chronic pain	Postoperative pain^A20^	- 2017	48	88, 75, 29, 21, 19, 15	-

^A1-A26^ corresponds to uptake study listed in Supplementary File C.

a Includes RCTs and observational studies.

b Assessed all 4 inner core domains plus 1 middle core domain.

c Uptake of 1 outcome not reported individually but included in full uptake assessment.

d Study included 2 COS.

e Excluded trials from assessment if they did not report at least 1 patient reported outcome (PRO).

f In 2009.

g SMARD.

h DC-ART.

i Excluded trials from assessment if they did not report at least 1 core outcome.

j Includes quasi-RCTs (3 acute, 2 chronic).

k 3 domains assessed as 1 domain not defined at time of review.

l Average from 2005 to 2018.

m In 2018.

**Table 2:** Characteristics of the COS assessed

COS disease category	COS disease name	Year COS published	No. outcomes in COS
Rheumatology	Rheumatoid arthritis	1982 1989 1994	10 7 7 + 1 (≥1 year)
	Knee, hip, and hand osteoarthritis	1997	3 + 1 (≥1 year)^a^
	Ankylosing spondylitis	1997	6 (SMARD) 9 (DC-ART)
	Acute and chronic gout	2005	5 (acute) 9 (chronic)
		2009	5 (acute)
	Psoriatic arthritis	2007	6
Anesthesia & pain control	Chronic pain	2003 2008 (update)	6 3
		2008	19
	Pediatric acute and chronic pain	2008	6
Orthopedics &	Fall injury	2005	5
Trauma	Spinal cord injury	2007	1
	Hip fracture	2014	5
Neurology	Peripheral neuropathy	2006	3
Skin	Eczema	2011	4

a 4 inner core domains, 2 middle core and 3 and others outer core.

### 3.3 Methods used to assess uptake

Seventeen (65%) studies identified the RCTs or SRs they would assess by carrying out a systematic literature review [[11], [12], [13], [14], [15], [16], [17], [18], [19], [20], [21], [22], [23], [24], [25], [26], [27]]. They extracted data about the outcomes included from the RCT reports and SRs. Two (8%) studies searched SRs to identify RCTs [28,29], and one (4%) study included RCTs identified from one systematic review [30]. Five (19%) studies identified RCTs by searching in a clinical trials registry [[31], [32], [33], [34], [35]]. One (4%) study identified RCTs through the citations received by the COS that they assessed and estimated the total number of RCTs as a denominator [36]. Twenty-one studies (81%) reviewed outcomes measured by their selected RCTs or SRs before the COS was published, or from the year of publication, as well as after. For those that only assessed the outcomes measured after the publication of the COS, the COS had been published for at least 3 years before the start of the uptake assessment period.

### 3.4 Uptake of the COS in full by RCTs and SRs

**Fig. 3:**
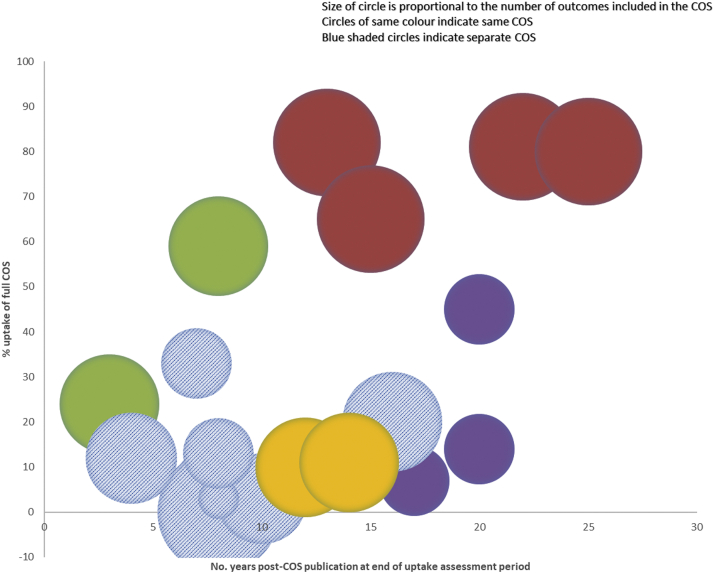
Uptake of full COS in RCTs and SRs.

Seventeen studies reported the proportion of RCTs that measured the full set of outcomes recommended by a COS, and one study reported this for SRs (Table 1). For four of the eight remaining studies, uptake assessment was not their main aim, and the other four studies did not indicate why they had not assessed uptake of the complete COS. For RCTs, the lowest rate of uptake reported was 0% (gout) and the highest 82% (rheumatoid arthritis), and 10% uptake was found by the study assessing SRs. Eleven of the COS had at least one study assessing uptake of the COS in full, and for eight of these (73%), at least one such study reported that a maximum of 20% or less of the RCTs or SRs assessed had measured the full COS. The assessed COS had recommended between one and 19 outcomes (Table 2). The COS with the least number of outcomes (*n* = 1) had an uptake rate of 3% of RCTs measuring the full COS. No RCT measured the full COS with the highest number of outcomes (*n* = 19), implicit from the fact that some of the outcomes were not measured in any RCT. The COS with the highest level of uptake recommended seven outcomes (plus one extra outcome for RCTs lasting more than 1 year) (Figure 3).

### 3.5 Uptake of individual outcomes in the COS by RCTs and SRs

Nineteen studies of RCTs reported the uptake of each outcome recommended by the COS, as did both studies of SRs (Table 1). The results showed a wide variation in the uptake rate for the individual outcomes in each COS. For example, one of those studies, assessing uptake of a COS for chronic pain, found that one outcome (pain) was included in 99% of trials while another (interpersonal functioning) was included in only 7% [21]. The authors of five studies suggested that a review of the COS may be needed to address this and one study planned to use its findings to update the COS, which was for psoriatic arthritis (PsA) [12]. Six outcomes out of a total of 133 across all studies of uptake in RCTs were reported by 100% of RCTs, and two of the six were from the same COS. None of the RCTs in one study for chronic gout measured three of the outcomes in the COS (which had nine COS outcomes in total), and none of the RCTs in a study for chronic pain measured 5 of the outcomes in the COS (19 outcomes in total).

### 3.6 Suggested barriers to uptake of COS

One of the studies investigated reasons for lack of uptake with the trialists directly [28] and reported that most of the trialists not measuring the full COS were not aware of it when designing their trial. A further 15 studies suggested potential barriers that may have resulted in low uptake of the COS (Table 3). The absence of validated measures, or no consensus on which instruments should be used to assess the domains, was noted in four studies [11,25,26,29]. Six studies referred to a limited patient or other key stakeholder involvement in the development of the COS as a potential barrier to uptake. Other barriers suggested were poor understanding of COS amongst trialists, lack of clarity, patient burden, cost, and lack of standardized recommendations across regulatory agencies.

**Table 3:** Suggested barriers to uptake of COS

Reason for low uptake	Number (%) of studies mentioning this reason	Example
Lack of awareness	5 (19)	“This appears to be associated with the lack of awareness of the researchers regarding the existence of this standardized set of outcomes.”^A21^
Lack of validated measures/no consensus on measures	4 (15)	“There may also be applicability issues due to a lack of consensus regarding instruments to assess each domain.”^A4^
Lack of patient involvement	4 (15)	“Further work is needed to obtain a better insight into what is relevant to the patient….”^A2^
Limited stakeholder involvement	2 (8)	“…the limited stakeholder involvement in the development of the hip fracture core outcome set may undermine its fitness for purpose.”^A24^
Poor understanding of COS	2 (8)	“…authors may not understand the purpose of core sets….”^A7^
Lack of clarity	1 (4)	“Precise definition of PsA Core Domains is necessary….”^A13^
Patient burden	1 (4)	“Patients, for instance, may experience the requirement to complete these measures as an onerous burden….”^A10^
Cost	1 (4)	“Previous research suggests some trialists do not measure damage as it is costly to measure and requires further expenditure to obtain valid readings of radiographs.”^A22^
Lack of standardized recommendations across regulatory agencies	1 (4)	“Some of this discordance may account for lack of uptake, and therefore future work may be undertaken to standardize recommendations across regulatory authorities.”^A23^

^A^ corresponds to the uptake study listed in Supplementary File C.

## 4 Discussion

There are currently few studies of COS uptake. The studies we identified covered five disease categories, with just over half of the uptake assessments being carried out for COS that had been developed for rheumatic diseases. Rheumatology has the second-highest number of published COS, and another two of the five disease categories with the most published COS (neurology and orthopedics and trauma) had at least one study assessing uptake of COS in its area [5]. We did not find any studies assessing the uptake of COS for cancer that has the highest number of COS of all disease categories [5]. For the remaining 25 disease categories that have at least one published COS, we did not find an assessment of uptake of any COS in these categories.

The studies included in our review used various methods to assess the uptake of COS. Most examined reports of RCTs that they had identified by reviewing the literature or searching systematic reviews. Not only are these lengthy processes, but also the information about the outcomes measured is not current as the outcomes would likely have been chosen some years before the trial reports were published. One study identified RCTs from the citations received by the COS publication. However, a previous study into citation analysis as a method for COS uptake assessment found that not all RCTs using a COS cite the COS publication [37]. A third method used, which removes the need to examine the report of the RCT and provides up to date information about the outcomes being measured, involved extracting information about outcomes from a trial registry. One of the uptake studies assessing the RA COS [31] tested this approach using ClinicalTrials.gov. The authors concluded that the uptake rate obtained by using the information listed in the registry alone (77%) was an acceptable estimate of the uptake rate found by identifying the RCTs on the registry and examining the results in the registry or report of the RCT (81%). This approach provides an efficient method to assess uptake, which may encourage further assessments to be carried out.

The studies that found low uptake of COS observed a number of barriers that might have hampered their use:

(i) To address a lack of patient and other key stakeholder involvement, and the issue of the relevance of outcomes in existing COS, it may be prudent to consider an update to the COS. The importance of patient involvement in COS development is being recognized by developers of new COS, with 94% of ongoing COS developers who responded to a 2017 survey stating that they had included patient participants [38]. While patient involvement may not in itself affect COS uptake, the relevance of COS will be improved with input from patient representation. Involving a range of key stakeholders when developing COS in addition to patients, for example, healthcare professionals, researchers, and those who might use the COS, may further improve the relevance of the outcomes selected for inclusion.
(ii) To tackle uncertainty around instruments and measures, COS developers should focus on determining *how* to measure the outcomes in the COS once consensus has been reached on *what* to measure. The COMET and COSMIN (COnsensus-based Standards for the selection of health Measurement Instruments) Initiatives have developed guidance on selecting measurement instruments for COS to aid developers in this process [39].
(iii) We did not observe any relationship between the number of outcomes recommended by a COS and its rate of uptake. However, in a survey about the uptake of the PedIMMPACT COS for pediatric acute and chronic pain, some authors of systematic reviews felt that the six domains in the COS were too many [40]. It is possible, however, that it is the perceived burden on patients to complete the measures that lead to reluctance to implement them, as noted by Mulla et al. in their study of the uptake of the IMMPACT COS for chronic pain [21]. COS developers may consider restricting the outcomes that are deemed to be core to a certain number, but in doing so, need to consider the risk of missing a critical measurement from the core set. COS developers should bear in mind the burden on both patients and healthcare professionals when considering outcomes and their measurement instruments.

Several studies compared the use of the outcomes that the COS recommended before and after the publication of the COS [17,19,29]. Only one of these studies, which assessed a COS for ankylosing spondylitis, noted some increase in uptake of the full COS after publication (0% RCTs before versus 20% RCTs after). The survey investigating the uptake of the PedIMMPACT COS found a lack of awareness of the COS, with only a third of authors of trials and systematic reviews who completed the survey being aware of the COS [40]. Lack of awareness was cited as an issue by a report of a similar survey for the IMMPACT COS for chronic pain [41]. The surveys also found that responders indicated that a lack of information about COS, lack of resources and time needed to use the COS, and in the case of systematic reviewers, the failure of RCTs to measure the COS outcomes, would affect the use of the COS. Difficulty in implementing the outcomes due to them being complicated was also noted. For investigating barriers and facilitators to COS uptake in more detail, a qualitative study is currently underway by the first author of this report, in which trialists are taking part in interviews.

We identified studies of COS uptake from the citation reports of COS publications. A limitation of our study is that it is possible that there are studies of uptake that did not cite the COS that they were assessing, and these would not have been identified in our search; however, we consider this to be unlikely.

Various strategies have been put in place to raise awareness of COS and encourage uptake. A set of minimum standards for COS development, COS-STAD [42], has been published to guide COS developers in producing high-quality COS and to give trialists considering a COS a benchmark against which to assess its quality. For improving accessibility to COS, the COMET Initiative’s database provides a freely accessible resource that collates all COS publications and allows researchers to identify potentially relevant COS for their study. Further strategies to consider in raising awareness include encouraging professional bodies to advocate for and promote COS, for example, through the inclusion of the topic in educational programs for researchers. A recent survey of developers of published COS, carried out by the COMET Initiative indicated that future studies of uptake are planned.

Overall, the studies that had assessed uptake of a COS in full found low levels of uptake. However, the standout exceptions to this were studies assessing uptake of the World Health Organisation and International League of Associations for Rheumatology COS for rheumatoid arthritis (RA). The four studies assessing this COS have shown consistently high levels of uptake from 60 to 70% of RCTs measuring the full COS in one study, to 82% of RCTs in another. In their 2013 assessment of the RA COS, Kirkham et al. suggested that this may be attributed in part to the endorsement of the COS by the Food and Drug Administration in 1996 and European Medicines Agency in 1998, after which they observed an increase in uptake [28]. In Kirkham’s subsequent review in 2017, it is noted that over 80% of trials assessed received commercial funding and so would have followed EMA/FDA guidance, including about the COS [31]. In their third update of this work in 2019, the authors found that the industry-funded trials were more likely to measure the COS [32]. This might suggest that endorsement by drug regulatory agencies improves the uptake of COS in RCTs, but, in contrast, a study that not only found a lower rate of uptake (45%) for the Knee, Hip, and Hand Osteoarthritis (OA) COS, also reported a decrease in its uptake over time, and noted some inconsistency in recommendations across regulators, which may have impacted on the uptake of the COS [33].

Some trial funders recommend the use of COS to their applicants (http://www.comet-initiative.org/COSEndorsement). A study assessed the impact of the National Institute for Health Research Health Technology Assessment’s (NIHR HTA) recommendation about COS and found that 38% of applicants who submitted a researcher-led bid for funding between January 2012 and December 2015 searched for a COS [43]. While the study concluded that trial funders could have an impact on COS uptake, it recommended further steps to increase this. Similar studies are ongoing for the Health Research Board (HRB) and Deutsche Forschungsgemeinschaft, German Research Foundation (DFG). There is a need for ongoing evaluations of such system-level recommendations to identify what works, what does not, and why.

With increased awareness of the need for COS and greater endorsement by influential organizations, we expect there to be more studies assessing COS uptake in the future. This review will serve as a benchmark for comparing uptake going forward.

## 5 Conclusions

To date, few studies have assessed uptake of COS in RCTs and SRs, and further work is needed to assess this across a wider range of health and COS areas and to understand the barriers and facilitators for uptake.

## CRediT authorship contribution statement

**Karen L. Hughes:** Conceptualization, Methodology, Data curation, Formal analysis, Investigation, Writing - original draft. **Mike Clarke:** Conceptualization, Methodology, Writing - review & editing, Supervision. **Paula R. Williamson:** Conceptualization, Methodology, Writing - review & editing, Supervision.

## Appendix A Supplementary data

Data Profile
