# Supplementary material for: A systematic review finds Core Outcome Set uptake varies widely across different areas of health
Source: J Clin Epidemiol. 2021 Jan;129:114–23. doi: 10.1016/j.jclinepi.2020.09.029 (PMC7815247; doi:10.1016/j.jclinepi.2020.09.029)
Supplement: Supplementary File A [file mmc2.docx]

**Supplementary File A: COS publications for citation analysis**

1. Dixon DO, McLaughlin P, Hagemeister FB, Freireich EJ, Fuller LM, et al. (1987) Reporting outcomes in Hodgkin's disease and lymphoma. Journal of Clinical Oncology 5: 1670-1672

2. Glynne-Jones R, Mawdsley S, Pearce T, Buyse M (2006) Alternative clinical end points in rectal cancer--are we getting closer? Annals of Oncology 17: 1239-1248

3. Auvinen A, Rietbergen JB, Denis LJ, Schroder FH, Prorok PC (1996) Prospective evaluation plan for randomised trials of prostate cancer screening. The International Prostate Cancer Screening Trial Evaluation Group. Journal of Medical Screening 3: 97-104

4. Denis L, Norlén BJ, Holmberg L, Begg CB, Damber JE, et al. (1997) Planning controlled clinical trials. Urology 49: 15-26

5. Scher HI, Eisenberger M, D'Amico AV, Halabi S, Small EJ, et al. (2004) Eligibility and outcomes reporting guidelines for clinical trials for patients in the state of a rising prostate-specific antigen: recommendations from the Prostate-Specific Antigen Working Group.[Erratum appears in J Clin Oncol. 2004 Aug 1;22(15):3205]. Journal of Clinical Oncology 22: 537-556

6. Dawson NA (1998) Apples and oranges: building a consensus for standardized eligibility criteria and end points in prostate cancer clinical trials. Journal of Clinical Oncology 16: 3398-3405

7. Middleton RG, Thompson IM, Austenfeld MS, Cooner WH, Correa RJ, et al. (1995) Prostate Cancer Clinical Guidelines Panel Summary report on the management of clinically localized prostate cancer. The American Urological Association. Journal of Urology 154: 2144-2148

8. Schellhammer P, Cockett A, Boccon-Gibod L, Gospodarowicz M, Krongrad A, et al. (1997) Assessment of endpoints for clinical trials for localized prostate cancer. Urology 49: 27-38

9. Rajkumar SV, Harousseau J-L, Durie B, Anderson KC, Dimopoulos M, et al. (2011) Consensus recommendations for the uniform reporting of clinical trials: report of the International Myeloma Workshop Consensus Panel 1. Blood 117: 4691-4695

10. Party IBMCW (2001) International bone metastases consensus on endpoint measurements for future clinical trials: proceedings of the first survey and meeting (work in progress) International Bone Metastases Consensus Working Party. Clinical Oncology (Royal College of Radiologists) 13: 82-84

11. Chow E, Wu JSY, Hoskin P, Coia LR, Bentzen SM, et al. (2002) International consensus on palliative radiotherapy endpoints for future clinical trials in bone metastases. Radiotherapy & Oncology 64: 275-280

12. Partsch H, Stout N, Forner-Cordero I, Flour M, Moffatt C, et al. (2010) Clinical trials needed to evaluate compression therapy in breast cancer related lymphedema (BCRL). Proposals from an expert group. International Angiology 29: 442-453

13. Hesketh PJ, Gralla RJ, du Bois A, Tonato M (1998) Methodology of antiemetic trials: response assessment, evaluation of new agents and definition of chemotherapy emetogenicity. Supportive Care in Cancer 6: 221-227

14. Micke O, Seegenschmiedt MH, German Working Group on Radiotherapy in G (2002) Consensus guidelines for radiation therapy of benign diseases: a multicenter approach in Germany. International Journal of Radiation Oncology, Biology, Physics 52

15. McVie JG, de Bruijn KM (1992) Methodology of antiemetic trials. Drugs 43 Suppl 3: 1-5

16. Pallis AG, Ring A, Fortpied C, Penninckx B, Van Nes MC, et al. (2011) EORTC workshop on clinical trial methodology in older individuals with a diagnosis of solid tumors. Annals of Oncology 22: 1922-1926

17. Miller AB, Hoogstraten B, Staquet M, Winkler A (1981) Reporting results of cancer treatment. Cancer 47: 207-214

18. Prorok PC, Marcus PM (2010) Cancer screening trials: nuts and bolts. Seminars in Oncology 37: 216-223

19. Punt CJA, Buyse M, Kohne C-H, Hohenberger P, Labianca R, et al. (2007) Endpoints in adjuvant treatment trials: a systematic review of the literature in colon cancer and proposed definitions for future trials. Journal of the National Cancer Institute 99: 998-1003

20. Wils J, Sahmoud T, Sobrero A, Bleiberg H, Ahmedzai S, et al. (1998) Evaluation of clinical efficacy of new medical treatments in advanced colorectal cancer. Results of a workshop organized by the EORTC GITCCG. European Organization for Research and Treatment of Cancer. Gastrointestinal Tract Cancer Cooperative Group. Tumori 84: 335-347

21. Llovet JM, Di Bisceglie AM, Bruix J, Kramer BS, Lencioni R, et al. (2008) Design and endpoints of clinical trials in hepatocellular carcinoma. Journal of the National Cancer Institute 100: 698-711

22. Pagliusi SR, Teresa Aguado M (2004) Efficacy and other milestones for human papillomavirus vaccine introduction. Vaccine 23: 569-578

23. Lefebvre J-L, Ang KK, Larynx Preservation Consensus P (2009) Larynx preservation clinical trial design: key issues and recommendations-a consensus panel summary. International Journal of Radiation Oncology, Biology, Physics 73: 1293-1303

24. Adelstein DJ, Ridge JA, Brizel DM, Holsinger FC, Haughey BH, et al. (2012) Transoral resection of pharyngeal cancer: summary of a National Cancer Institute Head and Neck Cancer Steering Committee Clinical Trials Planning Meeting, November 6-7, 2011, Arlington, Virginia. Head & Neck 34: 1681-1703

25. Gridelli C, Ardizzoni A, Le Chevalier T, Manegold C, Perrone F, et al. (2004) Treatment of advanced non-small-cell lung cancer patients with ECOG performance status 2: results of an European Experts Panel. Annals of Oncology 15: 419-426

26. Gridelli C, de Marinis F, Di Maio M, Ardizzoni A, Belani CP, et al. (2012) Maintenance treatment of advanced non-small-cell lung cancer: Results of an International Expert Panel Meeting of the Italian Association of Thoracic Oncology. Lung Cancer 76: 269-279

27. Cheson BD, Horning SJ, Coiffier B, Shipp MA, Fisher RI, et al. (1999) Report of an international workshop to standardize response criteria for non-Hodgkin's lymphomas. NCI Sponsored International Working Group. Journal of Clinical Oncology 17: 1244

28. Cheson BD, Pfistner B, Juweid ME, Gascoyne RD, Specht L, et al. (2007) Revised response criteria for malignant lymphoma. Journal of Clinical Oncology 25: 579-586

29. Anderson KC, Kyle RA, Rajkumar SV, Stewart AK, Weber D, et al. (2008) Clinically relevant end points and new drug approvals for myeloma. Leukemia 22: 231-239

30. Cheson BD, Bennett JM, Kopecky KJ, Buchner T, Willman CL, et al. (2003) Revised recommendations of the International Working Group for Diagnosis, Standardization of Response Criteria, Treatment Outcomes, and Reporting Standards for Therapeutic Trials in Acute Myeloid Leukemia.[Erratum appears in J Clin Oncol. 2004 Feb 1;22(3):576 Note: LoCocco, Francesco [corrected to Lo-Coco, Francesco]]. Journal of Clinical Oncology 21: 4642-4649

31. Kulke MH, Siu LL, Tepper JE, Fisher G, Jaffe D, et al. (2011) Future directions in the treatment of neuroendocrine tumors: consensus report of the National Cancer Institute Neuroendocrine Tumor clinical trials planning meeting. Journal of Clinical Oncology 29: 934-943

32. Bellm LA, Cunningham G, Durnell L, Eilers J, Epstein JB, et al. (2002) Defining clinically meaningful outcomes in the evaluation of new treatments for oral mucositis: oral mucositis patient provider advisory board. Cancer Investigation 20: 793-800

33. du Bois A, Quinn M, Thigpen T, Vermorken J, Avall-Lundqvist E, et al. (2005) 2004 consensus statements on the management of ovarian cancer: final document of the 3rd International Gynecologic Cancer Intergroup Ovarian Cancer Consensus Conference (GCIG OCCC 2004). Annals of Oncology 16 Suppl 8: viii7-viii12

34. Stuart GCE, Kitchener H, Bacon M, duBois A, Friedlander M, et al. (2011) 2010 Gynecologic Cancer InterGroup (GCIG) consensus statement on clinical trials in ovarian cancer: report from the Fourth Ovarian Cancer Consensus Conference. International Journal of Gynecological Cancer 21: 750-755

35. Thigpen T, duBois A, McAlpine J, DiSaia P, Fujiwara K, et al. (2011) First-line therapy in ovarian cancer trials. International Journal of Gynecological Cancer 21: 756-762

36. Comenzo RL, Reece D, Palladini G, Seldin D, Sanchorawala V, et al. (2012) Consensus guidelines for the conduct and reporting of clinical trials in systemic light-chain amyloidosis. Leukemia 26: 2317-2325

37. Dorman S, Jolley C, Abernethy A, Currow D, Johnson M, et al. (2009) Researching breathlessness in palliative care: consensus statement of the National Cancer Research Institute Palliative Care Breathlessness Subgroup. Palliative Medicine 23: 213-227

38. Renal Disease Subcommittee of the American College of Rheumatology Ad Hoc Committee on Systemic Lupus Erythematosus Response C (2006) The American College of Rheumatology response criteria for proliferative and membranous renal disease in systemic lupus erythematosus clinical trials. Arthritis & Rheumatism 54: 421-432

39. Bertsias GK, Ioannidis JPA, Boletis J, Bombardieri S, Cervera R, et al. (2009) EULAR points to consider for conducting clinical trials in systemic lupus erythematosus: literature based evidence for the selection of endpoints. Annals of the Rheumatic Diseases 68: 477-483

40. Gordon C, Bertsias G, Ioannidis JPA, Boletis J, Bombardieri S, et al. (2009) EULAR points to consider for conducting clinical trials in systemic lupus erythematosus. Annals of the Rheumatic Diseases 68: 470-476

41. Smolen JS, Strand V, Cardiel M, Edworthy S, Furst D, et al. (1999) Randomized clinical trials and longitudinal observational studies in systemic lupus erythematosus: consensus on a preliminary core set of outcome domains. Journal of Rheumatology 26: 504-507

42. Ruperto N, Ravelli A, Murray KJ, Lovell DJ, Andersson-Gare B, et al. (2003) Preliminary core sets of measures for disease activity and damage assessment in juvenile systemic lupus erythematosus and juvenile dermatomyositis. Rheumatology 42: 1452-1459

43. White B, Bauer EA, Goldsmith LA, Hochberg MC, Katz LM, et al. (1995) Guidelines for clinical trials in systemic sclerosis (scleroderma). I. Disease-modifying interventions. The American College of Rheumatology Committee on Design and Outcomes in Clinical Trials in Systemic Sclerosis. Arthritis & Rheumatism 38: 351-360

44. Khanna D, Lovell DJ, Giannini E, Clements PJ, Merkel PA, et al. (2008) Development of a provisional core set of response measures for clinical trials of systemic sclerosis. Annals of the Rheumatic Diseases 67: 703-709

45. Clements PJ, Allanore Y, Khanna D, Singh M, Furst DE (2012) Arthritis in Systemic Sclerosis: Systematic Review of the Literature and Suggestions for the Performance of Future Clinical Trials in Systemic Sclerosis Arthritis. Seminars in Arthritis and Rheumatism 41: 801-814

46. Khanna D, Brown KK, Clements PJ, Elashoff R, Furst DE, et al. (2010) Systemic sclerosis-associated interstitial lung disease-proposed recommendations for future randomized clinical trials. Clinical & Experimental Rheumatology 28: S55-62

47. Merkel PA, Herlyn K, Mahr AD, Neogi T, Seo P, et al. (2009) Progress towards a core set of outcome measures in small-vessel vasculitis. Report from OMERACT 9. Journal of Rheumatology 36: 2362-2368

48. Merkel PA, Aydin SZ, Boers M, Direskeneli H, Herlyn K, et al. (2011) The OMERACT core set of outcome measures for use in clinical trials of ANCA-associated vasculitis. Journal of Rheumatology 38: 1480-1486

49. Hellmich B, Flossmann O, Gross WL, Bacon P, Cohen-Tervaert JW, et al. (2007) EULAR recommendations for conducting clinical studies and/or clinical trials in systemic vasculitis: focus on anti-neutrophil cytoplasm antibody-associated vasculitis. Annals of the Rheumatic Diseases 66: 605-617

50. Felson DT (1993) Choosing a core set of disease activity measures for rheumatoid arthritis clinical trials. Journal of Rheumatology 20: 531-534

51. Fried BJ, Boers M, Baker PR (1993) A method for achieving consensus on rheumatoid arthritis outcome measures: the OMERACT conference process. Journal of Rheumatology 20: 548-551

52. Tugwell P, Boers M (1993) Developing consensus on preliminary core efficacy endpoints for rheumatoid arthritis clinical trials. OMERACT Committee. Journal of Rheumatology 20: 555-556

53. Boers M, Tugwell P, Felson DT, van Riel PL, Kirwan JR, et al. (1994) World Health Organization and International League of Associations for Rheumatology core endpoints for symptom modifying antirheumatic drugs in rheumatoid arthritis clinical trials. Journal of Rheumatology - Supplement 41: 86-89

54. Kirwan J, Heiberg T, Hewlett S, Hughes R, Kvien T, et al. (2003) Outcomes from the Patient Perspective Workshop at OMERACT 6. Journal of Rheumatology 30: 868-872

55. Kirwan JR, Hewlett SE, Heiberg T, Hughes RA, Carr M, et al. (2005) Incorporating the patient perspective into outcome assessment in rheumatoid arthritis - progress at OMERACT 7. Journal of Rheumatology. pp. 2250-2256

56. Kirwan JR, Minnock P, Adebajo A, Bresnihan B, Choy E, et al. (2007) Patient perspective: fatigue as a recommended patient centered outcome measure in rheumatoid arthritis. Journal of Rheumatology 34: 1174-1177

57. Sanderson T, Morris M, Calnan M, Richards P, Hewlett S (2010) Patient perspective of measuring treatment efficacy: the rheumatoid arthritis patient priorities for pharmacologic interventions outcomes. Arthritis care & research 62: 647-656

58. Sanderson T, Morris M, Calnan M, Richards P, Hewlett S (2010) What outcomes from pharmacologic treatments are important to people with rheumatoid arthritis? Creating the basis of a patient core set. Arthritis care & research 62: 640-646

59. Bombardier C, Tugwell P, Sinclair A, Dok C, Anderson G, et al. (1982) Preference for endpoint measures in clinical trials: results of structured workshops. Journal of Rheumatology 9: 798-801

60. Scott DL, Spector TD, Pullar T, McConkey B (1989) What should we hope to achieve when treating rheumatoid arthritis? Annals of the Rheumatic Diseases 48: 256-261

61. van Riel PL (1992) Provisional guidelines for measuring disease activity in clinical trials on rheumatoid arthritis. Br J Rheumatol 31: 793-794

62. Taylor WJ (2005) Preliminary identification of core domains for outcome studies in psoriatic arthritis using Delphi methods. Annals of the Rheumatic Diseases 64 Suppl 2: ii110-112

63. Gladman DD, Strand V, Mease PJ, Antoni C, Nash P, et al. (2005) OMERACT 7 psoriatic arthritis workshop: synopsis. Annals of the Rheumatic Diseases 64 Suppl 2: ii115-116

64. Gladman DD (2005) Consensus exercise on domains in psoriatic arthritis. Annals of the Rheumatic Diseases 64 Suppl 2: ii113-114

65. Gladman DD, Mease PJ, Strand V, Healy P, Helliwell PS, et al. (2007) Consensus on a core set of domains for psoriatic arthritis. Journal of Rheumatology 34: 1167-1170

66. Miller FW, Rider LG, Chung YL, Cooper R, Danko K, et al. (2001) Proposed preliminary core set measures for disease outcome assessment in adult and juvenile idiopathic inflammatory myopathies. Rheumatology 40: 1262-1273

67. Van Der Heijde D, Bellamy N, Calin A, Dougados M, Khan MA, et al. (1997) Preliminary core sets for endpoints in ankylosing spondylitis. Journal of Rheumatology 24: 2225-2229

68. Wolfe F, Lassere M, van der Heijde D, Stucki G, Suarez-Almazor M, et al. (1999) Preliminary core set of domains and reporting requirements for longitudinal observational studies in rheumatology. Journal of Rheumatology 26: 484-489

69. Mease PJ, Clauw DJ, Arnold LM, Goldenberg DL, Witter J, et al. (2005) Fibromyalgia syndrome. Journal of Rheumatology 32: 2270-2277

70. Mease P, Arnold LM, Bennett R, Boonen A, Buskila D, et al. (2007) Fibromyalgia syndrome. Journal of Rheumatology 34: 1415-1425

71. Arnold LM, Crofford LJ, Mease PJ, Burgess SM, Palmer SC, et al. (2008) Patient perspectives on the impact of fibromyalgia. Patient Education & Counseling 73: 114-120

72. Mease PJ, Arnold LM, Crofford LJ, Williams DA, Russell IJ, et al. (2008) Identifying the clinical domains of fibromyalgia: contributions from clinician and patient Delphi exercises. Arthritis & Rheumatism 59: 952-960

73. Carville SF, Choy E H (2008) Systematic review of discriminating power of outcome measures used in clinical trials of fibrymyalgia. Journal of Rheumatology 35: 2094-2105

74. Choy EH, Arnold LM, Clauw DJ, Crofford LJ, Glass JM, et al. (2009) Content and criterion validity of the preliminary core dataset for clinical trials in fibromyalgia syndrome. Journal of Rheumatology 36: 2330-2334

75. Mease P, Arnold LM, Choy EH, Clauw DJ, Crofford LJ, et al. (2009) Fibromyalgia syndrome module at OMERACT 9: domain construct. Journal of Rheumatology 36: 2318-2329

76. Salaffi F, Ciapetti A, Sarzi Puttini P, Atzeni F, Iannuccelli C, et al. (2012) Preliminary identification of key clinical domains for outcome evaluation in fibromyalgia using the Delphi method: The Italian experience. Reumatismo 64: 27-34

77. Schumacher HR, Edwards, N L, Perez-Ruiz, F et al (2005) Outcome measures for acute and chronic gout. Journal of Rheumatology 32: 2452-2455

78. Schumacher HR, Taylor W, Joseph-Ridge N, Perez-Ruiz F, Chen LX, et al. (2007) Outcome evaluations in gout. Journal of Rheumatology 34: 1381-1385

79. Taylor WJ, Schumacher HR, Jr., Baraf HSB, Chapman P, Stamp L, et al. (2008) A modified Delphi exercise to determine the extent of consensus with OMERACT outcome domains for studies of acute and chronic gout.[Erratum appears in Ann Rheum Dis. 2008 Nov;67(11):1652. Note: Mellado, J Vazquez [corrected to Vazquez-Mellado, J]]. Annals of the Rheumatic Diseases 67: 888-891

80. Schumacher HR, Taylor W, Edwards L, Grainger R, Schlesinger N, et al. (2009) Outcome domains for studies of acute and chronic gout. Journal of Rheumatology 36: 2342-2345

81. Giannini EH, Ruperto N, Ravelli A, Lovell DJ, Felson DT, et al. (1997) Preliminary definition of improvement in juvenile arthritis. Arthritis & Rheumatism 40: 1202-1209

82. Bellamy N, Kirwan J, Boers M, Brooks P, Strand V, et al. (1997) Recommendations for a core set of outcome measures for future phase III clinical trials in knee, hip, and hand osteoarthritis. Consensus development at OMERACT III. Journal of Rheumatology 24: 799-802

83. Heiligenhaus A, Foeldvari I, Edelsten C, Smith JR, Saurenmann RK, et al. (2012) Proposed outcome measures for prospective clinical trials in juvenile idiopathic arthritis-associated uveitis: a consensus effort from the multinational interdisciplinary working group for uveitis in childhood. Arthritis care & research 64: 1365-1372

84. Bowman SJ, Pillemer S, Jonsson R, Asmussen K, Vitali C, et al. (2001) Revisiting Sjögren's syndrome in the new millennium: Perspectives on assessment and outcome measures. Report of a workshop held on 23 March 2000 at Oxford, UK. Rheumatology 40: 1180-1188

85. Pillemer SR, Smith J, Fox PC, Bowman SJ (2005) Outcome measures for Sjogren's syndrome, April 10-11, 2003, Bethesda, Maryland, USA. Journal of Rheumatology 32: 143-149

86. Cranney A, Tugwell P, Cummings S, Sambrook P, Adachi J, et al. (1997) Osteoporosis clinical trials endpoints: candidate variables and clinimetric properties. Journal of Rheumatology 24: 1222-1229

87. (1997) Guidelines for osteoporosis trials. Journal of Rheumatology 24: 1234-1236

88. Sjodahl Hammarlund C, Nilsson MH, Hagell P (2012) Measuring outcomes in Parkinson's disease: a multi-perspective concept mapping study. Quality of Life Research 21: 453-463

89. Vellas B, Andrieu S, Sampaio C, Coley N, Wilcock G, et al. (2008) Endpoints for trials in Alzheimer's disease: a European task force consensus. Lancet Neurology 7: 436-450

90. (1995) World Federation of Neurology Research Group on Neuromuscular Diseases Subcommittee on Motor Neuron Disease. Airlie House guidelines. Therapeutic trials in amyotrophic lateral sclerosis. Airlie House "Therapeutic Trials in ALS" Workshop Contributors. Journal of the Neurological Sciences 129 Suppl: 1-10

91. Miller RG, Munsat TL, Swash M, Brooks BR (1999) Consensus guidelines for the design and implementation of clinical trials in ALS. World Federation of Neurology committee on Research. Journal of the Neurological Sciences 169: 2-12

92. Leigh PN, Swash M, Iwasaki Y, Ludolph A, Meininger V, et al. (2004) Amyotrophic lateral sclerosis: a consensus viewpoint on designing and implementing a clinical trial. Amyotrophic Lateral Sclerosis & Other Motor Neuron Disorders 5: 84-98

93. Shankaran S, Laptook A (2003) Challenge of conducting trials of neuroprotection in the asphyxiated term infant. Seminars in Perinatology 27: 320-332

94. Vargus-Adams JN, Martin LK (2009) Measuring what matters in cerebral palsy: a breadth of important domains and outcome measures. Archives of Physical Medicine & Rehabilitation 90: 2089-2095

95. Katona C, Livingston G, Cooper C, Ames D, Brodaty H, et al. (2007) International Psychogeriatric Association consensus statement on defining and measuring treatment benefits in dementia. International Psychogeriatrics 19: 345-354

96. Moniz-Cook E, Vernooij-Dassen M, Woods R, Verhey F, Chattat R, et al. (2008) A European consensus on outcome measures for psychosocial intervention research in dementia care. Aging & Mental Health 12: 14-29

97. (1998) Considerations on designing clinical trials to evaluate the place of new antiepileptic drugs in the treatment of newly diagnosed and chronic patients with epilepsy. Epilepsia 39: 799-803

98. LaFrance WC, Jr., Alper K, Babcock D, Barry JJ, Benbadis S, et al. (2006) Nonepileptic seizures treatment workshop summary. Epilepsy & Behavior 8: 451-461

99. Osborne JP, Lux A (2001) Towards an international consensus on definitions and standardised outcome measures for therapeutic trials (and epidemiological studies) in West syndrome. Brain & Development 23: 677-682

100. Lux AL, Osborne JP (2004) A proposal for case definitions and outcome measures in studies of infantile spasms and West syndrome: consensus statement of the West Delphi group. Epilepsia 45: 1416-1428

101. Mindell JA, Emslie G, Blumer J, Genel M, Glaze D, et al. (2006) Pharmacologic management of insomnia in children and adolescents: consensus statement. Pediatrics 117: e1223-1232

102. Schumacher HC, Meyers PM, Higashida RT, Derdeyn CP, Lavine SD, et al. (2010) Reporting standards for angioplasty and stent-assisted angioplasty for intracranial atherosclerosis. Journal of Neurointerventional Surgery 2: 324-340

103. Whitaker JN, McFarland HF, Rudge P, Reingold SC (1995) Outcomes assessment in multiple sclerosis clinical trials: a critical analysis. Multiple Sclerosis 1: 37-47

104. Chitnis T, Tenembaum S, Banwell B, Krupp L, Pohl D, et al. (2012) Consensus statement: evaluation of new and existing therapeutics for pediatric multiple sclerosis. Multiple Sclerosis 18: 116-127

105. Chitnis T, Tardieu M, Amato MP, Banwell B, Bar-Or A, et al. (2013) International Pediatric MS Study Group Clinical Trials Summit: meeting report. Neurology 80: 1161-1168

106. Penzien DB (2005) Guidelines for trials of behavioral treatments for recurrent headache: purpose, process, and product. Headache 45 Suppl 2: S87-89

107. Penzien DB, Andrasik F, Freidenberg BM, Houle TT, Lake AE, 3rd, et al. (2005) Guidelines for trials of behavioral treatments for recurrent headache, first edition: American Headache Society Behavioral Clinical Trials Workgroup. Headache 45 Suppl 2: S110-132

108. Andrasik F, Lipchik GL, McCrory DC, Wittrock DA (2005) Outcome measurement in behavioral headache research: headache parameters and psychosocial outcomes. Headache 45: 429-437

109. (1991) Guidelines for controlled trials of drugs in migraine. First edition. International Headache Society Committee on Clinical Trials in Migraine. Cephalalgia 11: 1-12

110. Tfelt-Hansen P, Block G, Dahlof C, Diener HC, Ferrari MD, et al. (2000) Guidelines for controlled trials of drugs in migraine: second edition. Cephalalgia 20: 765-786

111. Tfelt-Hansen P, Pascual J, Ramadan N, Dahlof C, D'Amico D, et al. (2012) Guidelines for controlled trials of drugs in migraine: third edition. A guide for investigators. Cephalalgia 32: 6-38

112. Lipton RB, Micieli G, Russell D, Solomon S, Tfelt-Hansen P, et al. (1995) Guidelines for controlled trials of drugs in cluster headache. Cephalalgia 15: 452-462

113. Schoenen J (1995) Guidelines for trials of drug treatments in tension-type headache. First edition: International Headache Society Committee on Clinical Trials. Cephalalgia 15: 165-179

114. Bendtsen L, Bigal ME, Cerbo R, Diener HC, Holroyd K, et al. (2010) Guidelines for controlled trials of drugs in tension-type headache: second edition. Cephalalgia 30: 1-16

115. Hughes R, Cidp, group MMNt (2005) 129th ENMC International Workshop: Clinical Trials for Chronic Inflammatory Demyelinating Polyradiculoneuropathy and Multifocal Motor Neuropathy, 27th October 2004, Schiphol airport, The Netherlands. Neuromuscular Disorders 15: 321-325

116. Merkies ISJ, Lauria G (2006) 131st ENMC international workshop: selection of outcome measures for peripheral neuropathy clinical trials 10-12 December 2004, Naarden, The Netherlands. Neuromuscular Disorders 16: 149-156

117. Reilly MM, de Jonghe P, Pareyson D (2006) 136th ENMC International Workshop: Charcot-Marie-Tooth disease type 1A (CMT1A)8-10 April 2005, Naarden, The Netherlands. Neuromuscular Disorders 16: 396-402

118. Clifton GL, Hayes RL, Levin HS, Michel ME, Choi SC (1992) Outcome measures for clinical trials involving traumatically brain-injured patients: report of a conference. Neurosurgery 31: 975-978

119. Wilde EA, Whiteneck GG, Bogner J, Bushnik T, Cifu DX, et al. (2010) Recommendations for the use of common outcome measures in traumatic brain injury research. Archives of Physical Medicine & Rehabilitation 91: 1650-1660 e1617

120. Duncan PW, Jorgensen HS, Wade DT (2000) Outcome measures in acute stroke trials: a systematic review and some recommendations to improve practice. Stroke 31: 1429-1438

121. Schellinger PD, Bath PMW, Lees KR, Bornstein NM, Uriel E, et al. (2012) Assessment of additional endpoints for trials in acute stroke - what, when, where, in who? International Journal of Stroke 7: 227-230

122. Hoeper MM, Oudiz RJ, Peacock A, Tapson VF, Haworth SG, et al. (2004) End points and clinical trial designs in pulmonary arterial hypertension: clinical and regulatory perspectives. Journal of the American College of Cardiology 43: 48S-55S

123. Distler O, Behrens F, Pittrow D, Huscher D, Denton CP, et al. (2008) Defining appropriate outcome measures in pulmonary arterial hypertension related to systemic sclerosis: a Delphi consensus study with cluster analysis.[Erratum appears in Arthritis Rheum. 2008 Aug 15;59(8):1202]. Arthritis & Rheumatism 59: 867-875

124. Becker LB, Aufderheide TP, Geocadin RG, Callaway CW, Lazar RM, et al. (2011) Primary outcomes for resuscitation science studies: a consensus statement from the American Heart Association. Circulation 124: 2158-2177

125. Chiam PTL, Ruiz CE (2008) Percutaneous transcatheter aortic valve implantation: assessing results, judging outcomes, and planning trials: the interventionalist perspective. Jacc: Cardiovascular Interventions 1: 341-350

126. Leon MB, Piazza N, Nikolsky E, Blackstone EH, Cutlip DE, et al. (2011) Standardized endpoint definitions for transcatheter aortic valve implantation clinical trials: a consensus report from the Valve Academic Research Consortium. European Heart Journal 32: 205-217

127. Kappetein AP, Head SJ, Genereux P, Piazza N, van Mieghem NM, et al. (2012) Updated standardized endpoint definitions for transcatheter aortic valve implantation: the Valve Academic Research Consortium-2 consensus document (VARC-2). European Journal of Cardio-Thoracic Surgery 42: S45-60

128. Cutlip DE, Windecker S, Mehran R, Boam A, Cohen DJ, et al. (2007) Clinical end points in coronary stent trials: a case for standardized definitions. Circulation 115: 2344-2351

129. Simons M, Bonow RO, Chronos NA, Cohen DJ, Giordano FJ, et al. (2000) Clinical trials in coronary angiogenesis: issues, problems, consensus: An expert panel summary. Circulation 102: E73-86

130. Conte MS, Geraghty PJ, Bradbury AW, Hevelone ND, Lipsitz SR, et al. (2009) Suggested objective performance goals and clinical trial design for evaluating catheter-based treatment of critical limb ischemia. Journal of Vascular Surgery 50: 1462-1473.e1461-1463.

131. Timaran CH, McKinsey JF, Schneider PA, Littooy F (2011) Reporting standards for carotid interventions from the Society for Vascular Surgery. Journal of Vascular Surgery 53: 1679-1695.

132. Stout N, Partsch H, Szolnoky G, Forner-Cordero I, Mosti G, et al. (2012) Chronic edema of the lower extremities: international consensus recommendations for compression therapy clinical research trials. International Angiology 31: 316-329

133. Nedeltchev K, Pattynama PM, Biaminoo G, Diehm N, Jaff MR, et al. (2010) Standardized definitions and clinical endpoints in carotid artery and supra-aortic trunk revascularization trials. Catheterization & Cardiovascular Interventions 76: 333-344

134. Anderson HV, Weintraub WS, Radford MJ, Kremers MS, Roe MT, et al. (2013) Standardized cardiovascular data for clinical research, registries, and patient care: A report from the data standards workgroup of the national cardiovascular research infrastructure project. Journal of the American College of Cardiology 61: 1835-1846

135. Hausenloy DJ, Erik Botker H, Condorelli G, Ferdinandy P, Garcia-Dorado D, et al. (2013) Translating cardioprotection for patient benefit: Position paper from the Working Group of Cellular Biology of the Heart of the European Society of Cardiology. Cardiovascular Research 98: 7-27

136. Labs KH, Dormandy JA, Jaeger KA, Stuerzebecher C, Hiatt WR (1999) Trans-atlantic conference on clinical trial guidelines in PAOD (Peripheral arterial occlusive disease) clinical trial methodology. European Journal of Vascular & Endovascular Surgery 18: 253-265

137. Mitchell LG, Goldenberg NA, Male C, Kenet G, Monagle P, et al. (2011) Definition of clinical efficacy and safety outcomes for clinical trials in deep venous thrombosis and pulmonary embolism in children. Journal of Thrombosis & Haemostasis 9: 1856-1858

138. Steg PG, Huber K, Andreotti F, Arnesen H, Atar D, et al. (2011) Bleeding in acute coronary syndromes and percutaneous coronary interventions: position paper by the Working Group on Thrombosis of the European Society of Cardiology. European Heart Journal 32: 1854-1864

139. O'Connell JB, McCarthy PM, Sopko G, Filippatos GS, Pina IL, et al. (2009) Mechanical circulatory support devices for acute heart failure syndromes: considerations for clinical trial design. Heart Failure Reviews 14: 101-112

140. Higashida RT, Furlan AJ, Roberts H, Tomsick T, Connors B, et al. (2003) Trial design and reporting standards for intra-arterial cerebral thrombolysis for acute ischemic stroke.[Erratum appears in Stroke. 2003 Nov;34(11):2774]. Stroke 34: e109-137

141. Calkins H, Kuck KH, Cappato R, Brugada J, Camm AJ, et al. (2012) 2012 HRS/EHRA/ECAS expert consensus statement on catheter and surgical ablation of atrial fibrillation: recommendations for patient selection, procedural techniques, patient management and follow-up, definitions, endpoints, and research trial design. Journal of Interventional Cardiac Electrophysiology 33: 171-257

142. Kirchhof P, Auricchio A, Bax J, Crijns H, Camm J, et al. (2007) Outcome parameters for trials in atrial fibrillation: Executive summary - Recommendations from a consensus conference organized by the German Atrial Fibrillation Competence NETwork (AFNET) and the European Heart Rhythm Association (EHRA). European Heart Journal 28: 2803-2817

143. Buser DA, Tonetti M (1997) Clinical trials on implants in regenerated bone. Annals of Periodontology 2: 329-342

144. Page RC, DeRouen TA (1992) Design issues specific to studies of periodontitis. Journal of Periodontal Research 27: 395-404; discussion 412-396

145. Imrey PB, Chilton NW, Pihlstrom BL, Proskin HM, Kingman A, et al. (1994) Proposed guidelines for American Dental Association acceptance of products for professional, non-surgical treatment of adult periodontitis. Task Force on Design and Analysis in Dental and Oral Research. Journal of Periodontal Research 29: 348-360

146. Lightfoot WS, Hefti A, Mariotti A (2005) Using a Delphi panel to survey criteria for successful periodontal therapy in anterior teeth. Journal of Periodontology 76: 1508-1512

147. Lightfoot WS, Hefti A, Mariotti A (2005) Using a Delphi panel to survey criteria for successful periodontal therapy in posterior teeth. Journal of Periodontology 76: 1502-1507

148. Weber HP, Fiorellini JP, Jeffcoat MC (1997) Clinical trials on placement of implants in existing bone. Annals of Periodontology 2: 315-328

149. Cochran DL (1998) Report of the American Academy of Periodontology's Workshop on the Design and Conduct of Clinical Trials for Endosseous Dental Implants. Journal of Periodontology 69: 280-284

150. Tonetti M, Palmer R (2012) Clinical research in implant dentistry: Study design, reporting and outcome measurements: Consensus report of Working Group 2 of the VIII European Workshop on Periodontology. Journal of Clinical Periodontology 39: 73-80

151. Smaïl-Faugeron V, Fron Chabouis H, Durieux P, Attal JP, Muller-Bolla M, et al. (2013) Development of a Core Set of Outcomes for Randomized Controlled Trials with Multiple Outcomes - Example of Pulp Treatments of Primary Teeth for Extensive Decay in Children. PLoS ONE 8

152. Chilton NW, Fleiss JL (1986) Design and analysis of plaque and gingivitis clinical trials. J Clin Periodontol 13: 400-410

153. (1986) Guidelines for acceptance of chemotherapeutic products for the control of supragingival dental plaque and gingivitis. Council on Dental Therapeutics. J Am Dent Assoc 112: 529-532

154. Pitts NB, Stamm JW (2004) International Consensus Workshop on Caries Clinical Trials (ICW-CCT)--final consensus statements: agreeing where the evidence leads. J Dent Res 83 Spec No C: C125-128

155. Marshall JC, Vincent J-L, Guyatt G, Angus DC, Abraham E, et al. (2005) Outcome measures for clinical research in sepsis: a report of the 2nd Cambridge Colloquium of the International Sepsis Forum. Critical Care Medicine 33: 1708-1716

156. Goldstein B, Giroir B, Randolph A, International Consensus Conference on Pediatric S (2005) International pediatric sepsis consensus conference: definitions for sepsis and organ dysfunction in pediatrics. Pediatric Critical Care Medicine 6: 2-8

157. Wood MJ, Balfour H, Beutner K, Bruxelle J, Fiddian P, et al. (1995) How should zoster trials be conducted? Journal of Antimicrobial Chemotherapy 36: 1089-1101

158. Barlow GD, Lamping DL, Davey PG, Nathwani D (2003) Evaluation of outcomes in community-acquired pneumonia: a guide for patients, physicians, and policy-makers. The Lancet Infectious Diseases 3: 476-488

159. Spellberg B, Talbot GH, Brass EP, Bradley JS, Boucher HW, et al. (2008) Position paper: recommended design features of future clinical trials of antibacterial agents for community-acquired pneumonia. Clinical Infectious Diseases 47 Suppl 3: S249-265

160. Powers JH (2010) Recommendations for improving the design, conduct, and analysis of clinical trials in hospital-acquired pneumonia and ventilator-associated pneumonia. Clinical Infectious Diseases 51 Suppl 1: S18-28

161. Spellberg B, Talbot G, Infectious Diseases Society of A, American College of Chest P, American Thoracic S, et al. (2010) Recommended design features of future clinical trials of antibacterial agents for hospital-acquired bacterial pneumonia and ventilator-associated bacterial pneumonia. Clinical Infectious Diseases 51 Suppl 1: S150-170

162. McCracken GH, Sande MA, Lentnek A, Whitley RJ, Scheld WM (1992) Evaluation of new anti-infective drugs for the treatment of acute bacterial meningitis. Infectious Diseases Society of America and the Food and Drug Administration. Clinical Infectious Diseases 15 Suppl 1: S182-188

163. Alioum A, Dabis F, Dequae-Merchadou L, Haverkamp G, Hudgens M, et al. (2001) Estimating the efficacy of interventions to prevent mother-to-child transmission of HIV in breast-feeding population: Development of a consensus methodology. Statistics in Medicine 20: 3539-3556

164. Kirkby R, Calabrese C, Kaltman L, Monnier J, Herscu P (2010) Methodological considerations for future controlled influenza treatment trials in complementary and alternative medicine. Journal of Alternative & Complementary Medicine 16: 275-283

165. Nystrom PO, Bax R, Dellinger EP, Dominioni L, Knaus WA, et al. (1990) Proposed definitions for diagnosis, severity scoring, stratification, and outcome for trials on intraabdominal infection. Joint Working Party of SIS North America and Europe. World Journal of Surgery 14: 148-158

166. Cross H (2005) A Delphi consensus on criteria for contraindications, assessment indicators and expected outcomes related to tibialis posterior transfer surgery. International Journal of Leprosy & Other Mycobacterial Diseases 73: 13-21

167. Moorthy V, Reed Z, Smith PG, Efficacy WHOSGoMoMV (2007) Measurement of malaria vaccine efficacy in phase III trials: report of a WHO consultation. Vaccine 25: 5115-5123

168. Moorthy VS, Reed Z, Smith PG, Committee WHOMVA (2009) MALVAC 2008: Measures of efficacy of malaria vaccines in phase 2b and phase 3 trials--scientific, regulatory and public health perspectives. Vaccine 27: 624-628

169. Steeves JD, Lammertse D, Curt A, Fawcett JW, Tuszynski MH, et al. (2007) Guidelines for the conduct of clinical trials for spinal cord injury (SCI) as developed by the ICCP panel: clinical trial outcome measures. Spinal Cord 45: 206-221

170. Bombardier C (2000) Outcome assessments in the evaluation of treatment of spinal disorders: summary and general recommendations. Spine 25: 3100-3103

171. Deyo RA, Battie M, Beurskens AJ, Bombardier C, Croft P, et al. (1998) Outcome measures for low back pain research. A proposal for standardized use. Spine 23: 2003-2013

172. Devogelaer JP, Dreiser RL, Abadie E, Avouac B, Bouvenot G, et al. (2003) Guidelines for clinical studies assessing the efficacy of drugs for the management of acute low back pain. Clinical & Experimental Rheumatology 21: 691-694

173. Goldhahn J, Scheele WH, Mitlak BH, Abadie E, Aspenberg P, et al. (2008) Clinical evaluation of medicinal products for acceleration of fracture healing in patients with osteoporosis. Bone 43: 343-347

174. Lynch AD, Logerstedt DS, Grindem H, Eitzen I, Hicks GE, et al. (2015) Consensus criteria for defining 'successful outcome' after ACL injury and reconstruction: A Delaware-Oslo ACL cohort investigation. British Journal of Sports Medicine 2015; 49:335-342

175. Falder S, Browne A, Edgar D, Staples E, Fong J, et al. (2009) Core outcomes for adult burn survivors: a clinical overview. Burns 35: 618-641

176. Reneman MF, Beemster TT, Edelaar MJA, van Velzen JM, van Bennekom C, et al. (2013) Towards an ICF- and IMMPACT-Based Pain Vocational Rehabilitation Core Set in the Netherlands. Journal of Occupational Rehabilitation: 1-9

177. Lamb SE, Jorstad-Stein EC, Hauer K, Becker C, Prevention of Falls Network E, et al. (2005) Development of a common outcome data set for fall injury prevention trials: the Prevention of Falls Network Europe consensus. Journal of the American Geriatrics Society 53: 1618-1622

178. Cameron ID, Robinovitch S, Birge S, Kannus P, Khan K, et al. (2010) Hip protectors: recommendations for conducting clinical trials--an international consensus statement (part II). Osteoporosis International 21: 1-10

179. Smith MA, Leeder SR, Jalaludin B, Smith WT (1996) The asthma health outcome indicators study. Australian & New Zealand Journal of Public Health 20: 69-75

180. Reddel HK, Taylor DR, Bateman ED, Boulet LP, Boushey HA, et al. (2009) An official American Thoracic Society/European Respiratory Society statement: asthma control and exacerbations: standardizing endpoints for clinical asthma trials and clinical practice. Am J Respir Crit Care Med 180: 59-99

181. Busse WW, Morgan WJ, Taggart V, Togias A (2012) Asthma outcomes workshop: overview. Journal of Allergy & Clinical Immunology 129: S1-8

182. Fuhlbrigge A, Peden D, Apter AJ, Boushey HA, Camargo CA, Jr., et al. (2012) Asthma outcomes: exacerbations. Journal of Allergy & Clinical Immunology 129: S34-48

183. Akinbami LJ, Sullivan SD, Campbell JD, Grundmeier RW, Hartert TV, et al. (2012) Asthma outcomes: healthcare utilization and costs. Journal of Allergy & Clinical Immunology 129: S49-64

184. Szefler SJ, Wenzel S, Brown R, Erzurum SC, Fahy JV, et al. (2012) Asthma outcomes: biomarkers. Journal of Allergy & Clinical Immunology 129: S9-23

185. Tepper RS, Wise RS, Covar R, Irvin CG, Kercsmar CM, et al. (2012) Asthma outcomes: pulmonary physiology. Journal of Allergy & Clinical Immunology 129: S65-87

186. Cloutier MM, Schatz M, Castro M, Clark N, Kelly HW, et al. (2012) Asthma outcomes: Composite scores of asthma control. Journal of Allergy and Clinical Immunology 129: S24-S33

187. Krishnan JA, Lemanske Jr RF, Canino GJ, Elward KS, Kattan M, et al. (2012) Asthma outcomes: Symptoms. Journal of Allergy and Clinical Immunology 129: S124-S135

188. Wilson SR, Rand CS, Cabana MD, Foggs MB, Halterman JS, et al. (2012) Asthma outcomes: Quality of life. Journal of Allergy and Clinical Immunology 129: S88-S123

189. Sinha IP, Gallagher R, Williamson PR, Smyth RL (2012) Development of a core outcome set for clinical trials in childhood asthma: a survey of clinicians, parents, and young people. Trials 13: 103

190. Keim SM, Spaite DW, Maio RF, Garrison HG, Desmond JS, et al. (2004) Risk adjustment and outcome measures for out-of-hospital respiratory distress. Academic Emergency Medicine 11: 1074-1081

191. Canonica GW, Baena-Cagnani CE, Bousquet J, Bousquet PJ, Lockey RF, et al. (2007) Recommendations for standardization of clinical trials with Allergen Specific Immunotherapy for respiratory allergy. A statement of a World Allergy Organization (WAO) taskforce. Allergy 62: 317-324

192. Cazzola M, MacNee W, Martinez FJ, Rabe KF, Franciosi LG, et al. (2008) Outcomes for COPD pharmacological trials: from lung function to biomarkers. European Respiratory Journal 31: 416-469

193. (1994) Capri S. Recommendations for guidelines on clinical trials of mucoactive drugs in chronic bronchitis and chronic obstructive pulmonary disease. Task Group on Mucoactive Drugs. Chest 106: 1532-1537

194. Dent J, Kahrilas PJ, Vakil N, Van Zanten SV, Bytzer P, et al. (2008) Clinical trial design in adult reflux disease: a methodological workshop. Alimentary Pharmacology & Therapeutics 28: 107-126

195. Wirth S, Kelly D, Sokal E, Socha P, Mieli-Vergani G, et al. (2011) Guidance for clinical trials for children and adolescents with chronic hepatitis C. Journal of Pediatric Gastroenterology & Nutrition 52: 233-237

196. Bajaj JS, Cordoba J, Mullen KD, Amodio P, Shawcross DL, et al. (2011) Review article: the design of clinical trials in hepatic encephalopathy--an International Society for Hepatic Encephalopathy and Nitrogen Metabolism (ISHEN) consensus statement. Alimentary Pharmacology & Therapeutics 33: 739-747

197. Fekety R, DuPont HL, Cooperstock M, Corrado ML, Murray DM (1992) Evaluation of new anti-infective drugs for the treatment of antibiotic-associated colitis. Infectious Diseases Society of America and the Food and Drug Administration. Clinical Infectious Diseases 15 Suppl 1: S263-267

198. Griffiths AM, Otley AR, Hyams J, Quiros AR, Grand RJ, et al. (2005) A review of activity indices and end points for clinical trials in children with Crohn's disease. Inflammatory Bowel Diseases 11: 185-196

199. Laine L, Spiegel B, Rostom A, Moayyedi P, Kuipers EJ, et al. (2010) Methodology for randomized trials of patients with nonvariceal upper gastrointestinal bleeding: recommendations from an international consensus conference. American Journal of Gastroenterology 105: 540-550

200. Pimentel M, Talley NJ, Quigley EM, Hani A, Sharara A, et al. (2013) Report from the multinational irritable bowel syndrome initiative 2012. Gastroenterology 144: e1-5

201. Sanyal AJ, Brunt EM, Kleiner DE, Kowdley KV, Chalasani N, et al. (2011) Endpoints and clinical trial design for nonalcoholic steatohepatitis. Hepatology 54: 344-353

202. Rahn DD, Abed H, Sung VW, Matteson KA, Rogers RG, et al. (2011) Systematic review highlights difficulty interpreting diverse clinical outcomes in abnormal uterine bleeding trials. Journal of Clinical Epidemiology 64: 293-300

203. Meuleman C, Tomassetti C, D'Hooghe TM (2012) Clinical outcome after laparoscopic radical excision of endometriosis and laparoscopic segmental bowel resection. Current Opinion in Obstetrics & Gynecology 24: 245-252

204. Vincent K, Kennedy S, Stratton P (2010) Pain scoring in endometriosis: entry criteria and outcome measures for clinical trials. Report from the Art and Science of Endometriosis meeting. Fertility & Sterility 93: 62-67

205. Broder MS, Landow WJ, Goodwin SC, Brook RH, Sherbourne CD, et al. (2000) An agenda for research into uterine artery embolization: results of an expert panel conference. Journal of Vascular & Interventional Radiology 11: 509-515

206. Basson R, Berman J, Burnett A, Derogatis L, Ferguson D, et al. (2000) Report of the international consensus development conference on female sexual dysfunction: definitions and classifications. Journal of Urology 163: 888-893

207. Clayton AH, Dennerstein L, Fisher WA, Kingsberg SA, Perelman MA, et al. (2010) Standards for clinical trials in sexual dysfunction in women: research designs and outcomes assessment. Journal of Sexual Medicine 7: 541-560

208. Walker M, Toneatto T, Potenza MN, Petry N, Ladouceur R, et al. (2006) A framework for reporting outcomes in problem gambling treatment research: the Banff, Alberta Consensus. Addiction 101: 504-511

209. Del Boca FK, Darkes J (2007) Enhancing the validity and utility of randomized clinical trials in addictions treatment research: II. Participant samples and assessment. Addiction 102: 1194-1203

210. Donovan DM, Bigelow GE, Brigham GS, Carroll KM, Cohen AJ, et al. (2012) Primary outcome indices in illicit drug dependence treatment research: systematic approach to selection and measurement of drug use end-points in clinical trials. Addiction 107: 694-708

211. Angus DC, Carlet J, Brussels Roundtable P (2003) Surviving intensive care: a report from the 2002 Brussels Roundtable. Intensive Care Medicine 29: 368-377

212. Levine LA, Greenfield JM (2003) Establishing a standardized evaluation of the man with Peyronie's disease. International Journal of Impotence Research 15 Suppl 5: S103-112

213. Djurhuus JC, Norgaard JP, Hjalmas K (1997) What is an acceptable treatment outcome? Scandinavian Journal of Urology & Nephrology Supplementum 183: 75-77

214. Toozs-Hobson P, Freeman R, Barber M, Maher C, Haylen B, et al. (2012) An International Urogynecological Association (IUGA)/International Continence Society (ICS) joint report on the terminology for reporting outcomes of surgical procedures for pelvic organ prolapse. International Urogynecology Journal 23: 527-535

215. Porst H, Vardi Y, Akkus E, Melman A, Park NC, et al. (2010) Standards for clinical trials in male sexual dysfunctions. Journal of Sexual Medicine 7: 414-444

216. Pavletic SZ, Martin P, Lee SJ, Mitchell S, Jacobsohn D, et al. (2006) Measuring therapeutic response in chronic graft-versus-host disease: National Institutes of Health Consensus Development Project on Criteria for Clinical Trials in Chronic Graft-versus-Host Disease: IV. Response Criteria Working Group report. Biology of Blood & Marrow Transplantation 12: 252-266

217. Lassila R, Rothschild C, De Moerloose P, Richards M, Perez R, et al. (2005) Recommendations for postmarketing surveillance studies in haemophilia and other bleeding disorders. Haemophilia 11: 353-359

218. Rodeghiero F, Stasi R, Gernsheimer T, Michel M, Provan D, et al. (2009) Standardization of terminology, definitions and outcome criteria in immune thrombocytopenic purpura of adults and children: report from an international working group. Blood 113: 2386-2393

219. Turk DC, Dworkin RH, Allen RR, Bellamy N, Brandenburg N, et al. (2003) Core outcome domains for chronic pain clinical trials: IMMPACT recommendations. Pain 106: 337-345

220. Turk DC, Dworkin RH, Revicki D, Harding G, Burke LB, et al. (2008) Identifying important outcome domains for chronic pain clinical trials: an IMMPACT survey of people with pain. Pain 137: 276-285

221. McGrath PJ, Walco GA, Turk DC, Dworkin RH, Brown MT, et al. (2008) Core outcome domains and measures for pediatric acute and chronic/recurrent pain clinical trials: PedIMMPACT recommendations. Journal of Pain 9: 771-783

222. Apfel C, Eberhart L, Kranke P, Ruesch D (2002) Recommendations for randomized controlled trials to prevent or treat postoperative nausea and vomiting. Anaesth Intensivmed Notfallmed Schmerzther 43: 69-74

223. Anderson JW, Pi-Sunyer FX, Danforth E, Dujovne CA, Greenway F, et al. (1998) Clinical trial design for obesity agents: a workshop report. Obesity Research 6: 311-315

224. Douglas RS, Tsirbas A, Gordon M, Lee D, Khadavi N, et al. (2009) Development of criteria for evaluating clinical response in thyroid eye disease using a modified Delphi technique. Archives of Ophthalmology 127: 1155-1160

225. Carlson GA, Jensen PS, Findling RL, Meyer RE, Calabrese J, et al. (2003) Methodological issues and controversies in clinical trials with child and adolescent patients with bipolar disorder: report of a consensus conference. Journal of Child & Adolescent Psychopharmacology 13: 13-27

226. Rush AJ, Kraemer HC, Sackeim HA, Fava M, Trivedi MH, et al. (2006) Report by the ACNP Task Force on response and remission in major depressive disorder. Neuropsychopharmacology 31: 1841-1853

227. Fitzpatrick R, Chambers J, Burns T, Doll H, Fazel S, et al. (2010) A systematic review of outcome measures used in forensic mental health research with consensus panel opinion. Health Technology Assessment (Winchester, England) 14: 1-94

228. Finer NN, Higgins R, Kattwinkel J, Martin RJ (2006) Summary proceedings from the apnea-of-prematurity group. Pediatrics 117: S47-51

229. Giacoia GP, Birenbaum DL, Sachs HC, Mattison DR (2006) The newborn drug development initiative. Pediatrics 117: S1-8

230. Short BL, Van Meurs K, Evans JR, Cardiology G (2006) Summary proceedings from the cardiology group on cardiovascular instability in preterm infants. Pediatrics 117: S34-39

231. Clancy RR (2006) Summary proceedings from the neurology group on neonatal seizures. Pediatrics 117: S23-S27

232. Gonzalez U, Whitton M, Eleftheriadou V, Pinart M, Batchelor J, et al. (2011) Guidelines for designing and reporting clinical trials in vitiligo. Archives of Dermatology 147: 1428-1436

233. Eleftheriadou V, Thomas KS, Whitton ME, Batchelor JM, Ravenscroft JC (2012) Which outcomes should we measure in vitiligo? Results of a systematic review and a survey among patients and clinicians on outcomes in vitiligo trials. British Journal of Dermatology 167: 804-814

234. Schmitt J, Langan S, Williams HC, European Dermato-Epidemiology N (2007) What are the best outcome measurements for atopic eczema? A systematic review. Journal of Allergy & Clinical Immunology 120: 1389-1398

235. Schmitt J, Williams H, Group HD (2010) Harmonising Outcome Measures for Eczema (HOME). Report from the First International Consensus Meeting (HOME 1), 24 July 2010, Munich, Germany. British Journal of Dermatology 163: 1166-1168

236. Schmitt J, Langan S, Stamm T, Williams HC, Harmonizing Outcome Measurements in Eczema Delphi p (2011) Core outcome domains for controlled trials and clinical recordkeeping in eczema: international multiperspective Delphi consensus process. Journal of Investigative Dermatology 131: 623-630

237. Schmitt J, Spuls P, Boers M, Thomas K, Chalmers J, et al. (2012) Towards global consensus on outcome measures for atopic eczema research: results of the HOME II meeting. Allergy 67: 1111-1117

238. Olliaro P, Vaillant M, Arana B, Grogl M, Modabber F, et al. (2013) Methodology of clinical trials aimed at assessing interventions for cutaneous leishmaniasis. PLoS Neglected Tropical Diseases [electronic resource] 7: e2130

239. Bellomo R, Ronco C, Kellum JA, Mehta RL, Palevsky P, et al. (2004) Acute renal failure - definition, outcome measures, animal models, fluid therapy and information technology needs: the Second International Consensus Conference of the Acute Dialysis Quality Initiative (ADQI) Group. Critical Care (London, England) 8: R204-212

240. Molitoris BA, Okusa MD, Palevsky PM, Kimmel PL, Star RA (2012) Designing clinical trials in acute kidney injury. Clinical Journal of the American Society of Nephrology 7: 842-843

241. Endre ZH, Pickering JW (2013) Acute kidney injury clinical trial design: old problems, new strategies. Pediatric Nephrology 28: 207-217

242. Abellan van Kan G, Cameron Chumlea W, Gillette-Guyonet S, Houles M, Dupuy C, et al. (2011) Clinical trials on sarcopenia: methodological issues regarding phase 3 trials. Clinics in Geriatric Medicine 27: 471-482

243. Devane D, Begley CM, Clarke M, Horey D, Oboyle C (2007) Evaluating maternity care: a core set of outcome measures. Birth 34: 164-172

244. Bennett WL, Robinson KA, Saldanha IJ, Wilson LM, Nicholson WK (2012) High priority research needs for gestational diabetes mellitus. Journal of Women's Health 21: 925-932

245. Langguth B, Goodey R, Azevedo A, Bjorne A, Cacace A, et al. (2007) Consensus for tinnitus patient assessment and treatment outcome measurement: Tinnitus Research Initiative meeting, Regensburg, July 2006. Progress in Brain Research 166: 525-536

246. Ramsey BW, Boat TF (1994) Outcome measures for clinical trials in cystic fibrosis. Summary of a Cystic Fibrosis Foundation consensus conference. Journal of Pediatrics 124: 177-192

247. Gottrup F, Apelqvist J, Price P, European Wound Management Association Patient Outcome G (2010) Outcomes in controlled and comparative studies on non-healing wounds: recommendations to improve the quality of evidence in wound management. Journal of Wound Care 19: 237-268

248. van Brussel M, van der Net J, Hulzebos E, Helders PJM, Takken T (2011) The Utrecht approach to exercise in chronic childhood conditions: the decade in review. Pediatric Physical Therapy 23: 2-14

249. Chen RC, Chang P, Vetter RJ, Lukka H, Stokes WA, Sanda MG, et al. Recommended patient-reported core set of symptoms to measure in prostate cancer treatment trials. J Natl Cancer Inst. 2014;106(7)

250. Chera BS, Eisbruch A, Murphy BA, Ridge JA, Gavin P, Reeve BB, et al. Recommended patient-reported core set of symptoms to measure in head and neck cancer treatment trials. J Natl Cancer Inst. 2014;106(7)

251. Fraser JF, Hussain MS, Eskey C, Abruzzo T, Bulsara K, English J, et al. Reporting standards for endovascular chemotherapy of head, neck and CNS tumors. J Neurointerv Surg. 2013;5(5):396-399

252. Donovan KA, Donovan HS, Cella D, Gaines ME, Penson RT, Plaxe SC, et al., Recommended patient-reported core set of symptoms and quality-of-life domains to measure in ovarian cancer treatment trials. J Natl Cancer Inst. 2014;106(7)

253. Glynne-Jones R, Adams RA, Jitlal M, Meadows H. End points in anal cancer: hopes for a common language. J Clin Oncol. 2014;32(12):1281-1282

254. Reeve BB, Mitchell SA, Dueck AC, Basch E, Cella D, Reilly CM, et al. Recommended patient-reported core set of symptoms to measure in adult cancer treatment trials. J Natl Cancer Inst. 2014;106(7)

255. Wildiers H, Mauer M, Pallis A, Hurria A, Mohile SG, Luciani A, et al. End points and trial design in geriatric oncology research: a joint European organisation for research and treatment of cancer--Alliance for Clinical Trials in Oncology--International Society Of Geriatric Oncology position article. J Clin Onco. 2013;31(29):3711-3718

256. Buch MH, Silva-Fernandez L, Carmona L, Aletah D, Christensen R, Combe B, et al. Development of EULAR recommendations for the reporting of clinical trial extension studies in rheumatology. Ann Rheum Dis. 2015;74:963-969

257. Ward L, Stebbings S, Sherman KJ, Cherkin D, Baxter GD, et al. Establishing key components of yoga interventions for musculoskeletal conditions: a Delphi survey. BMC Complement Altern Med. 2014;14:196

258. Kloppenburg M, Bøyesen P, Smeets W, Haugen IK, Liu R, Visser W, et al. Report from the omeract hand osteoarthritis special interest group: Advances and future research priorities. Rheumatol. 2014;41(4):810-818

259. Ball C, Pratt AL, Nanchahal J. Optimal functional outcome measures for assessing treatment for Dupuytren’s disease: a systematic review and recommendations for future practice. BMC Musculoskelet Disord. 2013;14:131

260. Goldhahn J, Beaton D, Ladd A, Macdermid J, Hoang-Kim A; Distal Radius Working Group of the International Society for Fracture Repair (ISFR); International Osteoporosis Foundation (IOF). Recommendation for measuring clinical outcome in distal radius fractures: a core set of domains for standardized reporting in clinical practice and research. Arch Orthop Trauma Surg. 2014;134(2):197-205

261. Haywood KL, Griffin XL, Achten J, Costa ML. Developing a core outcome set for hip fracture trials. Bone Joint J. 2014;96-B(8):1016-1023

262. Saketkoo LA, Mittoo S, Frankel S, LeSage D, Saver C, Phillips K, et al. Reconciling healthcare professional and patient perspectives in the development of disease activity and response criteria in connective tissue disease-related interstitial lung diseases. J Rheumatol. 2014;41(4):792-798

263. Saketkoo LA, Mittoo S, Huscher D, Khanna D, Dellaripa PF, Distler O, et al. Connective tissue disease related interstitial lung diseases and idiopathic pulmonary fibrosis: provisional core sets of domains and instruments for use in clinical trials. Thorax. 2014;69(5): 428-436

264. Spragg RG, Bernard GR, Checkley W, Curtis JR, Gajic O, Guyatt G, et al. Beyond Mortality - Future Clinical Research in Acute Lung Injury. Am J Respir Crit Care Med. 2010;181(10): 1121-1127

265. Eliasson AC, Krumlinde-Sundholm L, Gordon AM, Feys H, Klingels K, Aarts PB, et al. Guidelines for future research in constraint-induced movement therapy for children with unilateral cerebral palsy: an expert consensus. Dev Med Child Neurol. 2014;56(2):125-137

266. Smelt AF, Louter MA, Kies D, Blom J, Terwindt G, van der Heijden GJ, et al. What do patients consider to be the most important outcomes for effectiveness studies on migraine treatment? Results of a Delphi study. PLoS One. 2014;6;9(6):e98933

267. Fong F, Rogozinska E, Allotey J, Kempley S, Shah DK, Thangaratinam S., et al. Development of maternal and neonatal composite outcomes for trials evaluating management of late-onset pre-eclampsia. Hypertens Pregnancy. 2014;33(2):115-131

268. Smith CA, Betts D. The practice of acupuncture and moxibustion to promote cephalic version for women with a breech presentation: implications for clinical practice and research. Complement Ther Med. 2014;22(1):75-80

269. Diehm N, Vermassen F, van Sambeek MR; DEFINE Investigators. Standardized definitions and clinical endpoints in trials investigating endovascular repair of aortic dissections. Eur J Vasc Endovasc Surg. 2013;46(6):645-650

270. Zannad F, Garcia AA, Anker SD, Armstrong PW, Calvo G, Cleland JG, et al. Clinical outcome endpoints in heart failure trials: a European Society of Cardiology Heart Failure Association consensus document. Eur J Heart Fail. 2013;15(10): 1082-1094

271. Plotkin SR, Blakeley JO, Dombi E, Fisher MJ, Hanemann CO, Walsh KS, et al. Achieving consensus for clinical trials: the REiNS International Collaboration. Neurology. 2013;81(21 Suppl 1):S1-5

272. Wolters PL, Martin S, Merker VL, Gardner KL, Hingtgen CM, Tonsgard JH, et al. Patient-reported outcomes in neurofibromatosis and schwannomatosis clinical trials. Neurology. 2013;81(21 Suppl 1):S6-14

273. Ruemmele FM, Hyams JS, Otley A, Griffiths A, Kolho KL, Dias JA, et al. Outcome measures for clinical trials in paediatric IBD: An evidence-based, expert-driven practical statement paper of the paediatric ECCO committee. Gut. 2015;64(3):438-46

274. Samuel N, Carradice D, Wallace T, Smith GE, Chetter IC. Endovenous thermal ablation for healing venous ulcers and preventing recurrence. Cochrane Database Syst Rev. 2013;10:CD009494

275. Simpson RC, Thomas KS, Murphy R. Outcome measures for vulval skin conditions: a systematic review of randomized controlled trials. Br J Dermatol. 2013;169(3):494-501

276. Wylde V, MacKichan F, Bruce J, Gooberman-Hill R. Assessment of chronic post-surgical pain after knee replacement: Development of a core outcome set. Eur J Pain. 2015;19(5):611-20

277. Feldman LS, Lee L, Fiore J Jr. What outcomes are important in the assessment of Enhanced Recovery After Surgery (ERAS) pathways? Can J Anesth. 2015;62(2):120-30

278. Neville A, Lee L, Antonescu I, Mayo NE, Vassiliou MC, Fried GM, et al. Systematic review of outcomes used to evaluate enhanced recovery after surgery. Br J Surg. 2014;101(3):159-170

279. Chiu AK, Din N, Ai N. Standardising reported outcomes of surgery for intermittent exotropia--a systematic literature review." Strabismus. 2014;22(1):32-36

280. Cook RJ, Heddle NM. Clinical trials evaluating pathogen-reduced platelet products: methodologic issues and recommendations. Transfusion. 2013;53(8):1843-1855

281. Coolsen MM, Clermonts SH, van Dam RM, Winkens B, Malagó M, Fusai GK, et al. Development of a composite endpoint for randomized controlled trials in pancreaticoduodenectomy. World J Surg. 2014;38(6):1468-1475

282. Currie AC, Cahill R, Delaney CP, Faiz OD, Kennedy RH. International expert consensus on endpoints for full-thickness laparoendoscopic colonic excision. Surg Endosc. 2016;30(4):1497-1502

283. Haeusler GM, Phillips RS, Lehrnbecher T, Thursky KA, Sung L, Ammann RA. Core outcomes and definitions for pediatric fever and neutropenia research: A consensus statement from an international panel. Pediatr Blood Cancer. 2015; 62:483-489. doi: 10.1002/pbc.25335

284. Potter S, Holcombe C, Ward JA, Blazeby JM. Development of a core outcome set for research and audit studies in reconstructive breast surgery. Br J Surg. 2015;102(11):1360-1371

285. van den Bos W, Muller BG, Ahmed H, Bangma CH, Barret E, Crouzet S, et al. Focal therapy in prostate cancer: international multidisciplinary consensus on trial design. Eur Urol. 2014;65(6):1078-1083

286. van den Bos W, Muller BG, de Bruin DM, de Castro Abreu AL, Chaussy C, Coleman JA, et al. Salvage ablative therapy in prostate cancer: International multidisciplinary consensus on trial design. Urol Oncol. 2015;33(11):495.e1-7. doi: 10.1016/j.urolonc.2015.06.015. Epub 2015 Jul 29

287. Phillips K, Taylor A, Mease PJ, Simon LS, Conaghan PG, Choy EH, et al. Harmonizing pain outcome measures: Results of the pre-OMERACT meeting on partnerships for consensus on patient-important pain outcome domains between the cochrane musculoskeletal group and OMERACT. J Rheumatol. 2015;Aug 1. pii: jrheum.141386. [Epub ahead of print]

288. Rendas-Baum R, Bayliss M, Kosinski M, Raju A, Zwillich SH, Wallenstein GV, et al. Koncz T. Measuring the effect of therapy in rheumatoid arthritis clinical trials from the patient's perspective. Curr Med Res Opin. 2014;30(7):1391-1403

289. Singh JA, Dohm M, Sprowson AP, Wall PD, Richards BL, Gossec L, et al. Outcome domains and measures in total joint replacement clinical trials: Can we harmonize them? An OMERACT collaborative initiative. J Rheumatol. 2015;42(12):2496-2502

290. Balakrishnan K, Bauman N, Chun RH, Darrow DH, Grimmer JF, Perkins JA, et al. Standardized outcome and reporting measures in pediatric head and neck lymphatic malformations. Otolaryngol Head Neck Surg. 2015;152(5):948-53. doi: 10.1177/0194599815577602. Epub 2015 Mar 31

291. Senni M, Paulus WJ, Gavazzi A, Fraser AG, Díez J, Solomon SD, et al. New strategies for heart failure with preserved ejection fraction: the importance of targeted therapies for heart failure phenotypes. Eur Heart J. 2014;35(40):2797-2815. doi: 10.1093/eurheartj/ehu204. Epub 2014 Aug 7

292. Stone GW, Vahanian AS, Adams DH, Abraham WT, Borer JS, Bax JJ, et al. Clinical trial design principles and endpoint definitions for transcatheter mitral valve repair and replacement: part 1: clinical trial design principles: A consensus document from the mitral valve academic research consortium. Eur Heart J. 2015;36(29):1851-1877. doi: 10.1093/eurheartj/ehv281. Epub 2015 Jul 13

293. Harbin Consensus Conference Workshop Group; Conference Chairs, Legro RS, Wu X; Scientific Committee, Barnhart KT, Farquhar C, Fauser BC, Mol B. Improving the reporting of clinical trials of infertility treatments (IMPRINT): modifying the CONSORT statement. Hum Reprod. 2014;29(10):2075-82. doi: 10.1093/humrep/deu218. Epub 2014 Sep 12

294. van ʼt Hooft J1, Duffy JM, Daly M, Williamson PR, Meher S, Thom E, et al. A Core Outcome Set for Evaluation of Interventions to Prevent Preterm Birth. Obstet Gynecol;127(1):49-58. doi: 10.1097/AOG.0000000000001195

295. Jones L, Othman M, Dowswell T, Alfirevic Z, Gates S, Newburn M, et al. Pain management for women in labour: an overview of systematic reviews. Cochrane Database Syst Rev. 2012; (3):CD009234. doi: 10.1002/14651858.CD009234.pub2

296. Capstick R, Giele H. Interventions for treating fingertip entrapment injuries in children. Cochrane Database Syst Rev. 2014;(4):CD009808. DOI: 10.1002/14651858.CD009808.pub2

297. Chiarotto A, Deyo RA, Terwee CB, Boers M, Buchbinder R, Corbin TP, et al. Core outcome domains for clinical trials in non-specific low back pain. Eur Spine J. 2015;24(6):1127-42. doi: 10.1007/s00586-015-3892-3. Epub 2015 Apr 5

298. Paul L, Coote S, Crosbie J, Dixon D, Hale L, Holloway E, et al. Core outcome measures for exercise studies in people with multiple sclerosis: recommendations from a multidisciplinary consensus meeting. Mult Scler. 2014;20(12):1641-1650. doi: 10.1177/1352458514526944. Epub 2014 Mar 17

299. Karas J, Ashkenazi S, Guarino A, Lo Vecchio A, Shamir R, Vandenplas Y, et al. A core outcome set for clinical trials in acute diarrhoea. Arch Dis Child. 2015;100(4):359-63. doi: 10.1136/archdischild-2014-307403. Epub 2014 Nov 20

300. Billings FT, Shaw AD. Clinical trial endpoints in acute kidney injury. Nephron. 2014;127(1-4):89-93

301. Bruce I, Harman N, Williamson P, Tierney S, Callery P, Mohiuddin S, et al. The management of otitis media with effusion in children with cleft palate (mOMEnt): A feasibility study and economic evaluation. Health Technol Assess. 2015;19(68):1-374. doi: 10.3310/hta19680

302. Harman NL, Bruce IA, Kirkham JJ, Tierney S, Callery P, O'Brien K, et al. The Importance of Integration of Stakeholder Views in Core Outcome Set Development: Otitis Media with Effusion in Children with Cleft Palate. PLoS ONE. 2015;10(6):e0129514. doi:10.1371/journal.pone.0129514

303. Allard A, Fellowes A, Shilling V, Janssens A, Beresford B, Morris C. Key health outcomes for children and young people with neurodisability: qualitative research with young people and parents. BMJ Open. 2014;4:e004611

304. Janssens A, Williams J, Tomlinson R, Logan S, Morris C. Health outcomes for children with neurodisability: what do professionals regard as primary targets? Arch Dis Child. 2014;99(10):927–32

305. Morris C, Janssens A, Shilling V, Allard A, Fellowes A, Tomlinson R, et al. Meaningful health outcomes for paediatric neurodisability: Stakeholder prioritisation and appropriateness of patient reported outcome measures. Health Qual Life Outcomes. 2015;13:87. doi: 10.1186/s12955-015-0284-7

306. Crabb DW, Bataller R, Chalasani NP, Kamath PS, Lucey M, Mathurin P, et al. Standard Definitions and Common Data Elements for Clinical Trials in Patients With Alcoholic Hepatitis: Recommendation From the NIAAA Alcoholic Hepatitis Consortia. Gastroenterology. 2016;150(4):785-90

307. Deyo, R.A., et al., Report of the NIH Task Force on Research Standards for Chronic Low Back Pain. Journal of Pain. 2014;15(6):569-85

308. Fair C, Cuttance J, Sharma N, Maslow G, Wiener L, Betz C, et al. International and interdisciplinary identification of health care transition outcomes. JAMA Pediatrics. 2016;170(3):205-11

309. Gerritsen A, Jacobs M, Henselmans I, van Hattum J, Efficace F, Creemers GJ, et al. Developing a core set of patient-reported outcomes in pancreatic cancer: A Delphi survey. European journal of cancer. 2016;57:68-77

310. Helliwell T, Brouwer E, Pease CT, Hughes R, Hill CL, Neill LM, et al. Development of a Provisional Core Domain Set for Polymyalgia Rheumatica: Report from the OMERACT 12 Polymyalgia Rheumatica Working Group. The Journal of rheumatology. 2016;43(1):182-6

311. Ismail R, Azuara-Blanco A, Ramsay CR. Consensus on outcome measures for glaucoma effectiveness trials: Results from a delphi and nominal group technique approaches. Journal of Glaucoma. 2016;25(6):539-546

312. Khanna D, Mittoo S, Aggarwal R, Proudman SM, Dalbeth N, Matteson EL, et al. Connective Tissue Disease-associated Interstitial Lung Diseases (CTD-ILD) - Report from OMERACT CTD-ILD Working Group. The Journal of rheumatology. 2015; 42(11):2168-71

313. Maahs DM, Buckingham BA, Castle JR, Cinar A, Damiano ER, Dassau E, et al. Outcome measures for artificial pancreas clinical trials: A consensus report. Diabetes Care. 2016;39(7):1175-9

314. Myatt L, Redman CW, Staff AC, Hansson S, Wilson ML, Laivuori H, et al. Strategy for Standardization of Preeclampsia Research. Hypertension. 2014;63(6):1293-301

315. Noble AJ, Marson, AG. Which outcomes should we measure in adult epilepsy trials? The views of people with epilepsy and informal carers. Epilepsy & Behavior. 2016;59:105-110

316. Pinder RM, Brkljac M, Rix L, Muir L, Brewster M. Treatment of Scaphoid Nonunion: A Systematic Review of the Existing Evidence. The Journal of Hand Surgery. 2015;40(9):1797-1805

317. Askenazi DJ, Morgan C, Goldstein SL, et al. Strategies to improve the understanding of long-term renal consequences after neonatal acute kidney injury. Pediatr Res. 2016;79(3):502-508

318. Blencowe NS, Strong S, McNair AG, et al. Reporting of short-term clinical outcomes after esophagectomy: a systematic review. Ann Surg. 2012;255:658–666

319. Chalmers, J. R., E. Simpson, et al. (2016). "Report from the fourth international consensus meeting to harmonize core outcome measures for atopic eczema/dermatitis clinical trials (HOME initiative)." British Journal of Dermatology 175(1): 69-79

320. Schmitt, J., S. Langan, et al. (2013). "Assessment of clinical signs of atopic dermatitis: A systematic review and recommendation." Journal of Allergy and Clinical Immunology 132(6): 1337-1347

321. Benstoem C, Moza A, Autschbach R, et al. Evaluating outcomes used in cardiothoracic

surgery interventional research: a systematic review of reviews to develop a core outcome set.

Benedetto U, editor. PLoS One. 2015;10:e0122204

322. Christiansen JS, Backeljauw PF, Bidlingmaier M, et al. Growth Hormone Research Society perspective on the development of long-acting growth hormone preparations. Eur J Endocrinol. 2016;174(6):C1-8

323. D¨ohner H, Estey EH, Amadori S, et al. European LeukemiaNet. Diagnosis and management of acute myeloid leukemia in adults: recommendations from an international expert panel, on behalf of the European LeukemiaNet. Blood. 2010;115(3):453-474

324. Garcia-Cardenas V, Armour C, Benrimoj SI, et al. Pharmacists' interventions on clinical asthma outcomes: a systematic review. Eur Respir J. 2016;47(4):1134-1143

325. Kalyoncu U, Ogdie A, Campbell W, Bingham CO 3rd, de Wit M, Gladman DD, et al. Systematic literature review of domains assessed in psoriatic arthritis to inform the update of the psoriatic arthritis core domain set. RMD Open. 2016; 2:e000217

326. Kamat AM, Sylvester RJ, Böhle A, et al. Definitions, End Points, and Clinical Trial Designs for Non-Muscle-Invasive Bladder Cancer: Recommendations From the International Bladder Cancer Group. J Clin Oncol. 2016;34(16):1935-1944

327. Sharif MO, Tejani-Sharif A, Kenny K, et al. A systematic review of outcome measures used in clinical trials of treatment interventions following traumatic dental injuries. Dent Traumatol. 2015;31:422–8

328. Marrie RA, Miller A, Sormani MP, et al. Recommendations for observational studies of comorbidity in multiple sclerosis. Neurology. 2016;86(15):1446-1453

329. McNamara RL, Spatz ES, Kelley TA, et al. Standardized Outcome Measurement for Patients With Coronary Artery Disease: Consensus From the International Consortium for Health Outcomes Measurement (ICHOM). J AM Heart Assoc. 2015;4(5). pii: e001767

330. Sanderson, T., J. Kirwan, et al. (2016). "Item Development and Face Validity of the Rheumatoid Arthritis Patient Priorities in Pharmacological Interventions Outcome Measures." The Patient: Patient-Centered Outcomes Research 9(2): 103-115

331. Sharrock AE, Barker T, Yuen HM, et al. Management and closure of the open abdomen after damage control laparotomy for trauma. A systematic review and meta-analysis. Injury. 2016;47(2):296-306

332. Steutel NF, Benninga MA, Langendam MW, et al. Reporting outcome measures in trials of infant colic. J Pediatr Gastroenterol Nutr. 2014;59:341-346

333. Stoner MC, Calligaro KD, Chaer RA, et al. Reporting standards of the Society for Vascular Surgery for endovascular treatment of chronic lower extremity peripheral artery disease: Executive summary. J Vasc Surg. 2016;64(1):227-228

334. Turnbull AE, Rabiee A, Davis WE, et al. Outcome measurement in ICU survivorship research from 1970-2013: a scoping review of 425 publications. Crit Care Med. 2016;44(7):1267-1277

335. Howell M, Tong A, Wong G, et al. Important outcomes for kidney transplant recipients: a nominal group and qualitative study. Am J Kidney Dis. 2012;60(2):186-196

336. Howell M, Wong G, Turner RM, et al. The consistency and reporting of quality-of-life outcomes in trials of immunosuppressive agents in kidney transplantation: a systematic review and meta-analysis. Am J Kidney Dis. 2016;67:762-774

337. Acar C, Kleinjan GH, van den Berg NS, et al. Advances in sentinel node dissection in prostate cancer from a technical perspective. Int J Urol. 2015;22:898-909
