# Supplementary material for: A systematic review finds Core Outcome Set uptake varies widely across different areas of health
Source: J Clin Epidemiol. 2021 Jan;129:114–23. doi: 10.1016/j.jclinepi.2020.09.029 (PMC7815247; doi:10.1016/j.jclinepi.2020.09.029)
Supplement: Supplementary File B [file mmc3.docx]

**Supplementary File B: Key words**

For COS:

core

outcome(s)

endpoint(s)

measure(s)

measurement(s)

data(set)

set(s)

standardis/ze(d)(ing)(ation)

domain(s)

common

For uptake:

strategy(ies)

assess(ed)(ing)(ment/s)

implement(ed)(ation)(ing)

consensus

appropriate(ness)(ly)

recommend(ed)(ation/s)(ing)

challenge(s)

obstacle(s)

barrier(s)

faciliate(s)(ion)(ors)(ed)(ing)

adopt(ion)(ing)(ed)

uptake
