# Supplementary material for: A systematic review finds Core Outcome Set uptake varies widely across different areas of health
Source: J Clin Epidemiol. 2021 Jan;129:114–23. doi: 10.1016/j.jclinepi.2020.09.029 (PMC7815247; doi:10.1016/j.jclinepi.2020.09.029)
Supplement: Supplementary File C [file mmc4.docx]

**Supplementary File C: Included COS uptake studies**

1. Uptake study:

van der Heide A, Jacobs JWG, Dinant HJ, Bijlsma JWJ. The impact of endpoint measures in rheumatoid arthritis clinical trials. Seminars in Arthritis and Rheumatism 1992;21(5):287-94.

COS assessed:

Scott DL, Spector TD, Pullar T, McConkey B. What should we hope to achieve when treating rheumatoid arthritis? Annals of the Rheumatic Diseases 1989;48(3):256-61.

Bombardier C, Tugwell P, Sinclair A, Dok C, et al. Preference for endpoint measures in clinical trials: results of structured workshops. Journal of Rheumatology 1982;9(5):798-801.

1. Uptake study:

Kalyoncu U, Dougados M, Gossec L, Daurès JP. Reporting of patient-reported outcomes in recent trials in rheumatoid arthritis: A systematic literature review. Annals of the Rheumatic Diseases 2009;68(2):183-90.

COS assessed:

Boers M, Tugwell P, Felson DT, Van Riel PLCM, et al. World Health Organization and International League of Associations for Rheumatology core endpoints for symptom modifying antirheumatic drugs in rheumatoid arthritis clinical trials. Journal of Rheumatology 1994;21(SUPPL. 41):86-9

1. Uptake study:

Beale M, Cella M, de C. Williams AC. Comparing patients’ and clinician-researchers’ outcome choice for psychological treatment of chronic pain. Pain 2011;152(10):2283-6.

COS assessed:

Turk DC, Dworkin RH, Revicki D, Harding G, Burke LB, Cella D, Cleeland CS, Cowan P, Farrar JT, Hertz S, Max MB, Rappaport BA. Identifying important outcome domains for chronic pain clinical trials: an IMMPACT survey of people with pain. Pain 2008;137:276–85.

1. Uptake study:

Palominos PE, Gaujoux-Viala C, Fautrel B, Dougados M, et al. Clinical outcomes in psoriatic arthritis: A systematic literature review. Arthritis Care & Research 2012;64(3):397-406.

COS assessed:

Gladman DD, Mease PJ, Strand V, Healy P, Helliwell PS, Fitzgerald O, et al. Consensus on a core set of domains for psoriatic arthritis. J Rheumatol 2007;34:1167–70.

1. Uptake study:

Marks M, Schoones JW, Kolling C, Herren DB, et al. Outcome measures and their measurement properties for trapeziometacarpal osteoarthritis: a systematic literature review. 2013. p. 822-38.

COS assessed:

Bellamy N, Kirwan J, Boers M et al. Recommendations for a core set of outcome measures for future phase III clinical trials in knee, hip, and hand osteoarthritis. Consensus development at OMERACT III. J Rheumatol. 1997, 24: 799–802.

1. Uptake study:

Kirkham JJ, Boers M, Tugwell P, Clarke M, et al. Outcome measures in rheumatoid arthritis randomised trials over the last 50 years. Trials 2013;14(1).

COS assessed:

Boers M, Tugwell P, Felson DT, Van Riel PLCM, et al. World Health Organization and International League of Associations for Rheumatology core endpoints for symptom modifying antirheumatic drugs in rheumatoid arthritis clinical trials. Journal of Rheumatology 1994;21(SUPPL. 41):86-9

1. Uptake study:

Bautista-Molano W, Navarro-Compán V, Landewé RBM, Boers M, et al. How well are the ASAS/OMERACT core outcome sets for ankylosing spondylitis implemented in randomized clinical trials? A systematic literature review. Clinical Rheumatology 2014;33(9):1313-22.

COS assessed:

van der Heijde D, Bellamy N, Calin A et al (1997) Preliminary core sets for endpoints in ankylosing spondylitis. J Rheumatol 24:2225–9

1. Uptake study:

Dalbeth N, Zhong CS, Grainger R. Outcome Measures in Acute Gout: A Systematic Literature Review. 2014, (3), p. 558.

COS assessed:

Schumacher HR, Taylor W, Edwards L, Grainger R, Schlesinger N, Dalbeth N, et al. Outcome domains for studies of acute and chronic gout. J Rheumatol. 2009; 36:2342–5.

1. Uptake study:

Hann KEJ, McCracken LM. A systematic review of randomized controlled trials of Acceptance and Commitment Therapy for adults with chronic pain: Outcome domains, design quality, and efficacy. Journal of Contextual Behavioral Science 2014;3(4):217-27.

COS assessed:

Turk DC, Dworkin RH, Allen RR, Bellamy N, Brandenburg N, Carr DB, Cleeland C, et al. Core outcome domains for chronic pain clinical trials: IMMPACT recommendations. PAIN 2003; 106:337–45.

1. Uptake study:

Mulla SM, Maqbool A, Sivananthan L, Lopes LC, et al. Reporting of IMMPACT-recommended core outcome domains among trials assessing opioids for chronic non-cancer pain. Pain 2015;156(9):1615-9.

COS assessed:

Turk DC, Dworkin RH, Allen RR, Bellamy N, Brandenburg N, Carr DB, Cleeland C, et al. Core outcome domains for chronic pain clinical trials: IMMPACT recommendations. PAIN 2003; 106:337–45.

Turk DC, Dworkin RH, Revicki D, Harding G, Burke LB, Cella D, Cleeland CS, Cowan P, Farrar JT, Hertz S. Identifying important outcome domains for chronic pain clinical trials: an IMMPACT survey of people with pain. PAIN 2008;137:276–85.

1. Uptake study:

Araújo F, Cordeiro I, Ramiro S, Falzon L, et al. Outcomes assessed in trials of gout and accordance with OMERACT-proposed domains: a systematic literature review. Rheumatology (Oxford, England) 2015;54(6):981-93.

COS assessed:

Schumacher R, Edwards L, Perez-Ruiz F et al. Outcome measures for acute and chronic gout. J Rheumatol 2005;32:2452-5.

1. Uptake study:

Pruppers MHJ, Draak THP, Faber CG, Merkies ISJ, et al. Outcome measures in MMN revisited: Further improvement needed. Journal of the Peripheral Nervous System 2015;20(3):306-18.

COS assessed:

Merkies IS, Lauria G (2006). 131st ENMC International workshop: selection of outcome measures for peripheral neuropathy clinical trials 10–12 December 2004, Naarden, The Netherlands.

Neuromuscul Disord 16:149–156.

1. Uptake study:

Kalyoncu U, Ogdie A, Campbell W, Bingham CO, et al. Systematic literature review of domains assessed in psoriatic arthritis to inform the update of the psoriatic arthritis core domain set. BMJ Publishing Group; 2016.

COS assessed:

Gladman DD, Mease PJ, Strand V, et al. Consensus on a core set of domains for psoriatic arthritis. J Rheumatol 2007;34:1167–70.

1. Uptake study:

Copsey B, Hopewell S, Becker C, Cameron ID, et al. Appraising the uptake and use of recommendations for a common outcome data set for clinical trials: A case study in fall injury prevention. Trials 2016;17(1).

COS assessed:

Lamb SE, Jørstad‐Stein EC, Hauer K, Becker C. Development of a common outcome data set for fall injury prevention trials: the Prevention of Falls Network Europe consensus. J Am Geriatr Soc. 2005;53(9):1618–22.

1. Uptake study:

Stothers L, Nigro M, Tsang B, Lazare D, et al. An integrative review of standardized clinical evaluation tool utilization in anticholinergic drug trials for neurogenic lower urinary tract dysfunction. Spinal Cord 2016;54(12):1114-20.

COS assessed:

Steeves JD, Lammertse D, Curt A, Fawcett JW, Tuszynski MH, Ditunno JF et al. Guidelines for the conduct of clinical trials for spinal cord injury (SCI) as developed by the ICCP panel: clinical trial outcome measures. Spinal Cord 2007; 45: 206–221.

1. Uptake study:

Lange T, Rataj E, Kopkow C, Lützner J, et al. Outcome Assessment in Total Knee Arthroplasty: A Systematic Review and Critical Appraisal. Journal of Arthroplasty 2017;32(2):653-65.e1.

COS assessed:

Bellamy N, Kirwan J, Boers M et al. Recommendations for a core set of outcome measures for future phase III clinical trials in knee, hip, and hand osteoarthritis. Consensus development at OMERACT III. J Rheumatol. 1997, 24: 799–802.

1. Uptake study:

Kirkham JJ, Clarke M, Williamson PR. A methodological approach for assessing the uptake of core outcome sets using ClinicalTrials.gov: findings from a review of randomised controlled trials of rheumatoid arthritis. BMJ (Clinical Research Ed.) 2017;357:j2262-j.

COS assessed:

Boers M, Tugwell P, Felson DT, Van Riel PLCM, et al. World Health Organization and International League of Associations for Rheumatology core endpoints for symptom modifying antirheumatic drugs in rheumatoid arthritis clinical trials. Journal of Rheumatology 1994;21(SUPPL. 41):86-9

1. Uptake study:

Boric K, Jelicic Kadic A, Boric M, Zarandi‐Nowroozi M, et al. Outcome domains and pain outcome measures in randomized controlled trials of interventions for postoperative pain in children and adolescents. European Journal of Pain 2018.

COS assessed:

McGrath, P. J., Walco, G. A., et al. (2008). "Core outcome domains and measures for pediatric acute and chronic/recurrent pain clinical trials: PedIMMPACT recommendations." Journal of Pain 9(9): 771-783

1. Uptake study:

Dosenovic S, Kadic AJ, Jeric M, Boric M, et al. Efficacy and Safety Outcome Domains and Outcome Measures in Systematic Reviews of Neuropathic Pain Conditions. 2018. p. 674-84.

COS assessed:

Turk DC, Dworkin RH, Allen RR, Bellamy N, Brandenburg N, Carr DB, Cleeland C, et al. Core outcome domains for chronic pain clinical trials: IMMPACT recommendations. PAIN 2003; 106:337–45.

1. Uptake study:

Boric K, Dosenovic S, Kadic AJ, Boric M, et al. Efficacy and Safety Outcomes in Systematic Reviews of Interventions for Postoperative Pain in Children: Comparison Against the Recommended Core Outcome Set. 2018. p. 2316-21.

COS assessed:

McGrath, P. J., Walco, G. A., et al. (2008). "Core outcome domains and measures for pediatric acute and chronic/recurrent pain clinical trials: PedIMMPACT recommendations." Journal of Pain 9(9): 771-783

1. Uptake study:

Farag AM, Albuquerque R, Ariyawardana A, Chmieliauskaite M, et al. World Workshop in Oral Medicine VII: Reporting of IMMPACT-recommended outcome domains in randomized controlled trials of burning mouth syndrome: A systematic review. Oral Diseases 2019(S1):122.

COS assessed:

Turk DC, Dworkin RH, Allen RR, Bellamy N, Brandenburg N, Carr DB, Cleeland C, et al. Core outcome domains for chronic pain clinical trials: IMMPACT recommendations. PAIN 2003; 106:337–45.

1. Uptake study:

Kirkham JJ, Bracken M, Hind L, Pennington K, et al. Industry funding was associated with increased use of core outcome sets. 2019. p. 90-7.

COS assessed:

Boers M, Tugwell P, Felson DT, Van Riel PLCM, et al. World Health Organization and International League of Associations for Rheumatology core endpoints for symptom modifying antirheumatic drugs in rheumatoid arthritis clinical trials. Journal of Rheumatology 1994;21(SUPPL. 41):86-9

1. Uptake study:

Smith TO, Arden NK, Mansfield M, Hawker GA, et al. Uptake of the OMERACT-OARSI hip and knee osteoarthritis core outcome set: Review of randomized controlled trials from 1997 to 2017. Journal of Rheumatology 2019;46(8):976-80.

COS assessed:

Bellamy N, Kirwan J, Boers M et al. Recommendations for a core set of outcome measures for future phase III clinical trials in knee, hip, and hand osteoarthritis. Consensus development at OMERACT III. J Rheumatol. 1997, 24: 799–802.

1. Uptake study:

Smith TO, Collier T, Sheehan KJ, Sherrington C. uptake of the hip fracture core outcome set: analysis of 20 years of hip fracture trials. Age & Ageing 2019;48(4):595-8.

COS assessed:

Haywood KL, Griffin XL, Achten J, Costa ML. Developing a core outcome set for hip fracture trials. Bone Joint J 2014; 96-B: 1016 –23.

1. Uptake study:

Krsticevic M, Boric K, Dosenovic S, Dimcea DAM, et al. Outcome domains, outcome measures, and characteristics of randomized controlled trials testing nonsurgical interventions for osteoarthritis. Journal of Rheumatology 2020;47(1):126-31.

COS assessed:

Bellamy N, Kirwan J, Boers M et al. Recommendations for a core set of outcome measures for future phase III clinical trials in knee, hip, and hand osteoarthritis. Consensus development at OMERACT III. J Rheumatol. 1997, 24: 799–802.

1. Uptake study:

Vincent R, Chalmers JR, McWilliams C, Thomas KS, et al. Assessing uptake of the Harmonising Outcome Measures for Eczema (HOME) Core Outcome Set and Recommended Instruments. The British journal of dermatology 2020.

COS assessed:

Schmitt J, Spuls P, Boers M, et al. Towards global consensus on outcome measures for atopic eczema research: results of the HOME II meeting. Allergy 2012; 67:1111–1117.
